# Regulation of p110δ PI 3-Kinase Gene Expression

**DOI:** 10.1371/journal.pone.0005145

**Published:** 2009-04-09

**Authors:** Klaartje Kok, Gemma E. Nock, Elizabeth A. G. Verrall, Michael P. Mitchell, Daan W. Hommes, Maikel P. Peppelenbosch, Bart Vanhaesebroeck

**Affiliations:** 1 Centre for Cell Signalling, Institute of Cancer, Queen Mary University of London, Charterhouse Square, London, United Kingdom; 2 Department of Cell Biology, University Medical Centre Groningen, University of Groningen, Groningen, The Netherlands; 3 Bioinformatics and Biostatistics, Cancer Research UK London Research Institute, London, United Kingdom; 4 Department of Gastroenterology and Hepatology; Leiden University Medical Centre, Leiden, The Netherlands; Ordway Research Institute, United States of America

## Abstract

**Background:**

Despite an intense interest in the biological functions of the phosphoinositide 3-kinase (PI3K) signalling enzymes, little is known about the regulation of PI3K gene expression. This also applies to the leukocyte-enriched p110δ catalytic subunit of PI3K, an enzyme that has attracted widespread interest because of its role in immunity and allergy.

**Principal Findings:**

We show that p110δ expression is mainly regulated at the transcriptional level. In fibroblasts, lymphocytes and myeloid cells, p110δ gene transcription appears to be constitutive and not subject to acute stimulation. 5′RACE experiments revealed that p110δ mRNA transcripts contain distinct upstream untranslated exons (named exon -1, -2a, -2b, -2c and -2d), which are located up to 81 kb upstream of the translational start codon in exon 1. The levels of all the different p110δ transcripts are higher in leukocytes compared to non-leukocytes, with the p110δ transcript containing exon -2a most abundantly expressed. We have identified a highly conserved transcription factor (TF) binding cluster in the p110δ gene which has enhanced promoter activity in leukocytes compared to non-leukocytes. In human, this TF cluster is located immediately upstream of exon -2a whilst in mouse, it is located within exon -2a.

**Conclusion:**

This study identifies a conserved *PIK3CD* promoter region that may account for the predominant leukocyte expression of p110δ.

## Introduction

Phosphoinositide 3-kinases (PI3Ks) generate lipid second messengers that regulate a broad variety of cellular responses such as growth, cell cycle progression, differentiation, vesicular traffic and cell migration [1]. PI3K activity is critical in a wide variety of normal and pathological physiological responses, including immune regulation, metabolic control and cancer. However, despite the importance of this signalling system, very little is known about the regulation of PI3K gene expression under normal and disease conditions.

The PI3K family is divided into 3 classes [2]. Class I PI3Ks are acutely activated upon receptor stimulation and are heterodimers consisting of a p110 catalytic subunit in complex with a regulatory subunit. The class I PI3Ks are further subdivided into class IA and IB, depending on whether the catalytic subunit is in complex with an SH2-domain containing regulatory subunit (collectively called ‘p85’) or with the p101 or p84 regulatory subunits, which lack SH2 domains. Mammals have 3 class IA p110 catalytic subunits, p110α, p110β and p110δ, encoded by 3 distinct genes, *PIK3CA, PIK3CB* and *PIK3CD*, respectively. These p110 isoforms interact with p85, of which there are at least five different species, called p85α, p55α and p50α (encoded by the *PIK3R1* gene) and p85β and p55γ (encoded by *PIK3R2* and *PIK3R3*, respectively). p110γ is the only class IB PI3K catalytic subunit and occurs in complex with p101 [3], [4] or p84 [5], [6], which have no homology to p85. Class I PI3Ks can be activated by tyrosine kinases (p110α, p110δ) or GPCRs (p110β and p110γ) [1], [7]–[9].

Tissue distribution and the regulation of PI3K expression has recently been reviewed [10]. Whereas p110α and p110β appear to have a broad tissue distribution [11]–[15], p110δ is highly expressed in leukocytes [12], [13], [16], found at intermediate levels in neurons [17] and present at low levels in most other cell types [13], [18]. p110δ is also expressed at moderate levels in some cancer cells of non-leukocyte origin such as melanoma and breast cancer cells, often with large differences in expression levels in cell lines of the same tissue origin [18], for reasons that are unclear at the moment. Like p110δ, p110γ is highly enriched in leukocytes [19]–[21] but is also found at lower levels in other cell types such as cardiomyocytes [22]–[24], endothelial cells [25], pancreatic islets [26], [27] and smooth muscle cells [28].

Expression of the class IA catalytic isoforms can be altered during physiological and pathological processes, including differentiation (p110α and p110β) [29], regeneration (p110α) [30], [31] and hypertension (p110β and p110δ) [32]–[34]. PI3K expression, especially of p110α, is also very frequently increased in cancer. Insulin and nuclear receptor ligands can induce expression of the class I regulatory subunits [35]–[39]. Other documented mechanisms of p85 regulation are through the transcription factors (TF) STAT3 (p55α and p50α) [40], EBNA-2 (p55α) [41] and SREBP (p55γ) [42] and through targeted degradation of p85α and p85β by microRNAs [43], [44] (reviewed in [10]).

Three recent studies have identified a transcription regulatory region for the human p110α gene, *PIK3CA*. The *PIK3CA* locus gives rise to two alternative transcripts which each contain a distinct 5′ untranslated exon (exon -1b or -1a) that is spliced onto the first translated (ATG-containing) exon. The genomic position of these 5′ untranslated exons is about 50 kb upstream of the translation start site [45], [46]. TF binding sites for p53 [45]), FOXO3a [46]) and NF-κB [47] have been mapped in close proximity to the most 5′ untranslated exon (called exon -1b). Whereas p53 might inhibit transcription of p110α, evidence for a positive regulation by NF-κB and FOXO3a has been presented.

A promoter region for murine p110γ has also been identified [19]. Multiple transcriptional start sites exist for p110γ, resulting in transcripts with varying 5′ untranslated regions (5′UTRs), up to 874 bp in length. Analysis of the genomic p110γ DNA up to 1.2 kb upstream from the transcription start site revealed that the putative promoter region contains consensus sites for housekeeping TFs such as AP1 and SP1, as well as several putative binding sites for leukocyte-specific TFs [19]. Functional analysis of this p110γ putative promoter region revealed enhanced promoter activity in the U937 myeloid cell line compared to the HeLa epithelial cell line [19].

In this study, we have investigated the regulation of p110δ gene expression. We have documented that p110δ protein expression largely correlates with the level of p110δ mRNA in numerous cell types, indicating that p110δ expression is predominantly regulated at the level of transcription. We have found multiple mouse and human p110δ transcripts that contain distinct upstream untranslated exons, which we have named exon -1, -2a, -2b, -2c and -2d, located up to 81 kb upstream of the translational start codon in exon 1. Furthermore, we have identified a highly conserved TF-binding cluster that is located within mouse exon -2a and located immediately 5′ upstream of human exon -2a. This TF-binding cluster has enhanced promoter activity in leukocytes compared to non-leukocytes. Out of the 7 different TF binding sites in the TF-binding cluster, 4 are associated with regulation of haematopoiesis and expression of leukocyte-specific genes. These findings are the first to identify a *PIK3CD* promoter and offer a rationale for the leukocyte-enriched expression of p110δ.

## Results

### p110δ protein expression is not altered in fibroblasts, B-lymphocytes and myelomonocytic cells upon acute stimulation with various agonists

We first investigated whether p110δ expression can be induced by several acute cellular stimuli. In NIH-3T3 fibroblasts, which contain very low levels of endogenous p110δ compared to leukocytes, p110δ could not be induced by TNF, the proteasome inhibitor PS-341, UV irradiation, osmotic stress or the glucocorticoid dexamethasone (data not shown). p110δ protein levels were also unaffected during different phases of the cell cycle in these cells (data not shown). In B lymphocytes, p110δ expression was not affected by stimulation of the antigen receptor using anti-IgM antibodies. In U937 myelomonocytic cells, p110δ levels were unaltered by treatment with retinoic acid, in contrast to the p110γ protein which was induced effectively (data not shown), the latter in line with previously published data [48], [49]. Taken together, p110δ expression appears not to be regulated in an acute manner in response to extracellular stimuli, at least in the cell types and conditions investigated.

### Correlation between p110δ mRNA and protein levels in cell lines

**Figure 1:**
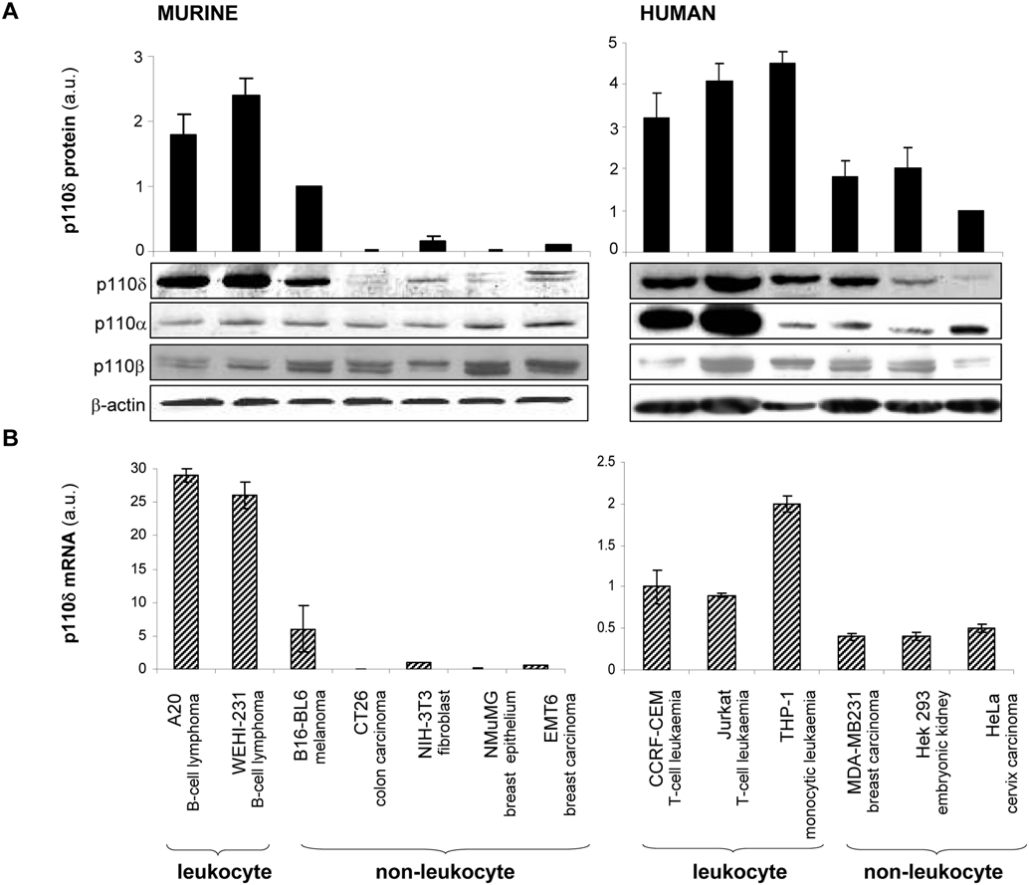
Correlation between p110δ protein and mRNA expression levels in murine and human cell lines. (A) Total cell lysates from the indicated cell lines were immunoblotted with antibodies to the distinct p110 isoforms or β-actin. One representative immunoblot of three independent experiments is shown. The bars represent quantification of the relative amounts of p110δ protein in mouse and human cell lines, as determined in 3 independent experiments (for each cell line, the ratio of the OD of the p110δ immunoblot signal was determined, relative to that of β-actin in this cell line. This value was then expressed relative to the p110δ/β-actin ratio found in B16-BL6 (for the mouse lines) or HeLa (for the human lines). Values are averages of three independent experiments. (B) Quantification of p110δ mRNA levels by real time RT-PCR using primers in the p110δ coding region. Signals are normalised to β-actin mRNA in each cell line. Data shown are the averages of three independent experiments.

We next assessed the levels of p110δ protein and mRNA, using immunoblotting of total cell lysates and real time RT-PCR, respectively, in a panel of murine and human cell lines (Figure 1A,B). p110δ mRNA and protein were found in all cell lines investigated but in widely varying amounts. In line with published data [12], [13], [16], [18], leukocytes expressed high levels of p110δ while non-leukocytes expressed intermediate to low levels. In line with previous data [12], a good correlation was found between p110δ mRNA and protein levels in most cell lines tested, indicating that p110δ protein expression is mainly regulated at the level of transcription.

### DNA methylation and histone acetylation are unlikely to be key mechanisms to control *PIK3CD* expression

**Figure 2:**
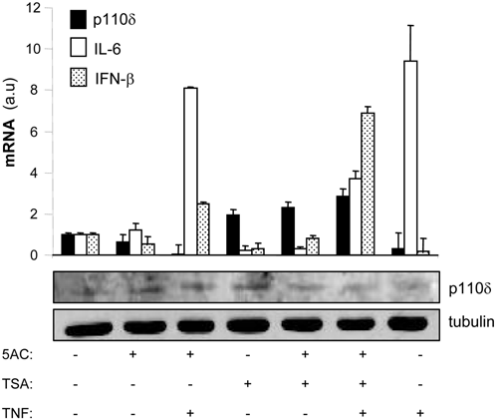
DNA methylation and histone acetylation do not alter p110δ expression in mouse L929 fibroblasts. (Top panel) L929 cells were treated with 5′-azacytidine (5AC,;5 µM) for 72 h and/or trichostatin A (TSA; 100 nM) for 6 h, with or without 6 h co-treatment with TNF (100 IU/ml)). mRNA levels of p110δ, IL-6 and IFN-β were quantified by real time RT-PCR. Samples were normalised for GAPDH and are relative to p110δ mRNA amounts in the unstimulated samples (set as 1). Shown is the average of three independent experiments). (Lower panel) Representative immunoblot (of three) of p110δ protein.

DNA methylation and histone acetylation are important epigenetic mechanisms that control gene expression by dictating transitions between transcriptionally active or transcriptionally silent chromatin states [50], [51]. L929 fibroblasts, which express low levels of p110δ mRNA and protein, were treated with 5′-azacytidine or trichostatin A, agents known to cause DNA (hemi-) demethylation and histone hyperacetylation, respectively, creating open configurations of genomic DNA to allow binding of TFs. As a positive control, we monitored the previously documented induction in these cells of mRNA expression of the cytokines IL-6 and IFN-β by 5′-azacytidine and trichostatin A [52], [53]. As can be seen from Figure 2, p110δ mRNA expression levels were not dramatically altered by any of these treatments, with a maximum increase in p110δ mRNA of around 2-fold, which was not accompanied by an induction of p110δ protein expression.

### The presence of high p110δ mRNA levels is not a consequence of leukocyte-specific p110δ mRNA stability

**Figure 3:**
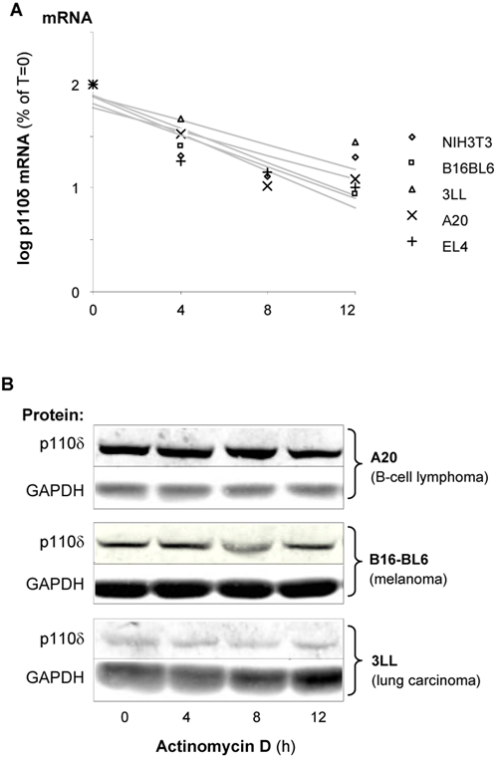
Equal p110δ mRNA stability in leukocytes and non-leukocytes. The indicated cell lines were treated with Actinomycin D (4 µg/ml), an inhibitor of *de novo* RNA synthesis, for the indicated time points followed by quantification of either p110δ mRNA (A) or p110δ protein (B). p110δ mRNA was quantified by real time RT-PCR, using normalisation for 18S RNA. p110δ mRNA levels are presented in a semi-log plot.

To assess whether high expression of the p110δ protein in cells is due to increased mRNA stability, cells were treated with Actinomycin D, an inhibitor of *de novo* RNA synthesis, followed by measurement of mRNA decay over time. As can be seen from Figure 3A, leukocyte (A20 and EL4) and non-leukocyte (B16-BL6, 3LL and NIH-3T3) cell lines displayed very similar rates of mRNA degradation upon inhibition of mRNA synthesis, indicating that there is no difference in p110δ mRNA stability between cell types expressing high or low levels of p110δ protein. Also p110δ protein levels were not affected by Actinomycin D treatment, both in leukocytes and non-leukocytes (Figure 3B).

### Identification of multiple distinct p110δ mRNA transcripts with alternate first 5′ untranslated exons

**Figure 4:**
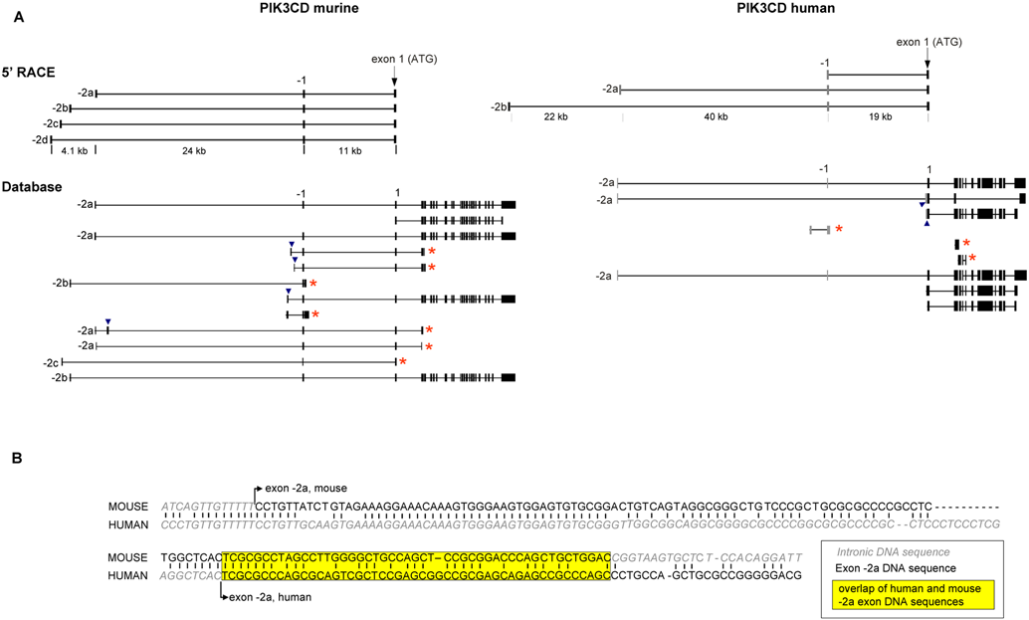
PIK3CD transcripts, assessed by 5′RACE and database analysis. (A) Top panel, Schematic representation of the different p110δ mRNA transcripts in their genomic context as found by 5′RACE in murine and human cell lines. Bottom panel, the different p110δ transcripts as found in the Ensembl database (release 52, 9 December 2008) for both species. Arrowheads indicate -2 exons present in the database which we have not found by 5′RACE. Asterisks indicate short *PIK3CD* transcripts (which do not encode full length p110δ protein) found in the Ensembl database. (B) Region of homology between mouse and human exon -2a. Exons 1 which contain the transcription start sites are indicated with a vertical arrow.

In order to identify the *PIK3CD* promoter, we set out to identify the transcriptional start site of the p110δ mRNA. Rapid amplification of 5′ cDNA ends (5′RACE) was used to identify the 5′UTR. BLAT alignment of the 5′RACE products led to three main observations: (1) multiple distinct p110δ transcripts exist within each cell line investigated; (2) most transcripts contains two untranslated exons (Figure 4A), which we have named exon -1 and -2 (to indicate their relative locations with respect to exon 1, which contains the putative ATG translation start site as defined in [13], [54]). The -1 and -2 exons are located 11 kb and >35 kb 5′ of exon 1 in murine cells, and 19 kb and >59 kb 5′ of exon 1 in human cells; (3) exon -1 can occur together with one of 4 identified second untranslated exons (exons -2a, -2b, -2c or -2d) in mouse cells, and with one of two -2 exons (-2a or -2b) in human cells. Thus, mouse and human *PIK3CD* can give rise to at least 4 and 2, respectively, distinct p110δ transcripts.

Human and mouse p110δ exon 1 contains an in-frame stop codon immediately upstream of the p110δ start codon (for sequences, see Supporting Information, File S1), ruling out the possibility that the newly identified upstream exons are translated as part of the p110δ protein. In other words, the -1 and -2 exons form the 5′UTR of the *PIK3CD* transcripts.

The -1 exons identified in human and mouse show a high degree of homology (data not shown) and likewise the -2a exons share a region of high homology (Figure 4B). Human exon -2a is approximately half the length of mouse exon -2a (72 bp compared to 144 bp), and its first part (1–49 bp) is homologous to the last part of mouse exon -2a (96–144 bp). Interestingly, the intronic DNA immediately upstream of human exon -2a is highly homologous with the first part of mouse exon -2a (Figure 4B). In contrast the -2b exons for human and mouse are not homologous, which may indicate that the -2b exon identified in human may not be the equivalent -2b exon identified in mouse.

Database information provides independent confirmation of several of the untranslated exons identified in this study (-1, -2a, -2b and -2c in the mouse; and -1, -2a in human), as well as additional -2 exons in the mouse (labeled with an arrowhead in Figure 4B) which we have thus far not found by 5′RACE both in mouse and human (>50 independent 5′RACE products sequenced; detailed data not shown).

The p110δ transcripts identified by 5′RACE always contained exon -1, and further incorporated a single -2 exon in all cases. We have found two instances where the -1/-2 exon of the p110δ mRNA configuration does not seem to occur. Firstly, during the cDNA cloning of human p110δ [13], we identified one clone (called o5) which did not contain exon -1, and which has exon -2a directly spliced onto exon 1, giving rise to a p110δ transcript that encodes full length p110δ protein (data not shown). Secondly, 2 out of 48 transcripts identified by 5′RACE in the mouse EL4 leukocyte cell line did not have -2 exons, and started with an exon -1. As shown in Table 1, all -2 and -1 exons contain a classical splice donor sequence (GT), whereas exon -1 only contains a splice acceptor sequence (AG). This is in line with the observation that each p110δ transcript identified by 5′RACE contains a single -2 exon and further indicates that the different p110δ transcripts arise individually and not from a ‘master’ p110δ transcript by alternative splicing. The presence of a splice acceptor sequence in exon -1 indicates that the p110δ transcript starting at this exon (as revealed by 5′RACE) found in mouse EL4 cells could in fact be an artefact, due to RNA degradation during the RACE experiments.

**Table 1:** Splice donor and acceptor sites in the 5′ introns/exons of *PIK3CD*.

Human						
Exon	Size (bp)	Splice acceptor	5′ end exon	3′ end exon	Splice donor	Intron (bp)
**-2b**	251	cgggggtca	GAGGCGCCCA	ACTCTGACAG	**gt**gagtcta	
						61,243
**-2a**	59	gcgcccagc	GCAGTCGCTC	CGCCGGGACG	**gt**aagcgat	
						39,665
**-1**	105	ccccaac**ag**	ATAAGGAGTC	TTCCAGAGAG	**gt**aggttgg	
						18,852
**1**	173	cattttt**ag**	GACAACTGTC	CATCAAGCAG	**gt**atggcct	
						4,944
**2**	229	tccctcc**ag**	CTGCTGTGGC	ATCGGCAAAG	**gt**agctctg	
Uppercase letters represent exon sequences, lowercase letters represent intron sequences.						
**Murine**						
**Exon**	**Size (bp)**	**Splice acceptor**	**5′ end exon**	**3′ end exon**	**Splice donor**	**Intron (bp)**
**-2d**	150	cttccgggc	TAGGACTTCT	GGAGCAGTTC	**gt**tttattta	
						28,348
**-2c**	78	gagagaga	ATCAGAAACC	CTACTCAAAT	**gt**cagattt	
						28,270
**-2b**	117	ttgagcggt	AAGAAAGCAG	ATGTAGAAGT	**gt**aagccaa	
						27,309
**-2a**	144	gttgttttt	CCTGTTATCT	TGCTGGACCG	**gt**aagtgct	
						24,360
**-1**	119	ttctttc**ag**	ACATCTAAGG	TACCAAACAG	**gt**aggttgg	
						10,759
**1**	173	ttcccac**ag**	GAAAACAGAC	CATCAAGCAG	**gt**agagcca	
						2,913
**2**	229	ctctccc**ag**	GTGCTGTGGC	ATTGGCAAAG	**gt**atactta	
Uppercase letters represent exon sequences, lowercase letters represent intron sequences.						

Splice donor and acceptor sites in p110δ exons. Splice acceptor and splice donor sequences of human (**top panel**) and murine (**lower panel**) p110δ exons. The untranslated exons as well as exons 1 and 2 are represented. Uppercase letters represent exon sequences, lowercase letters represent intron sequences. AG/GT splice donor/acceptor sequences are in bold. All other coding exons of p110δ follow the same AG/GT splicing rule (not shown).

### Cell type-specific usage of the multiple *PIK3CD* transcription start sites

**Figure 5:**
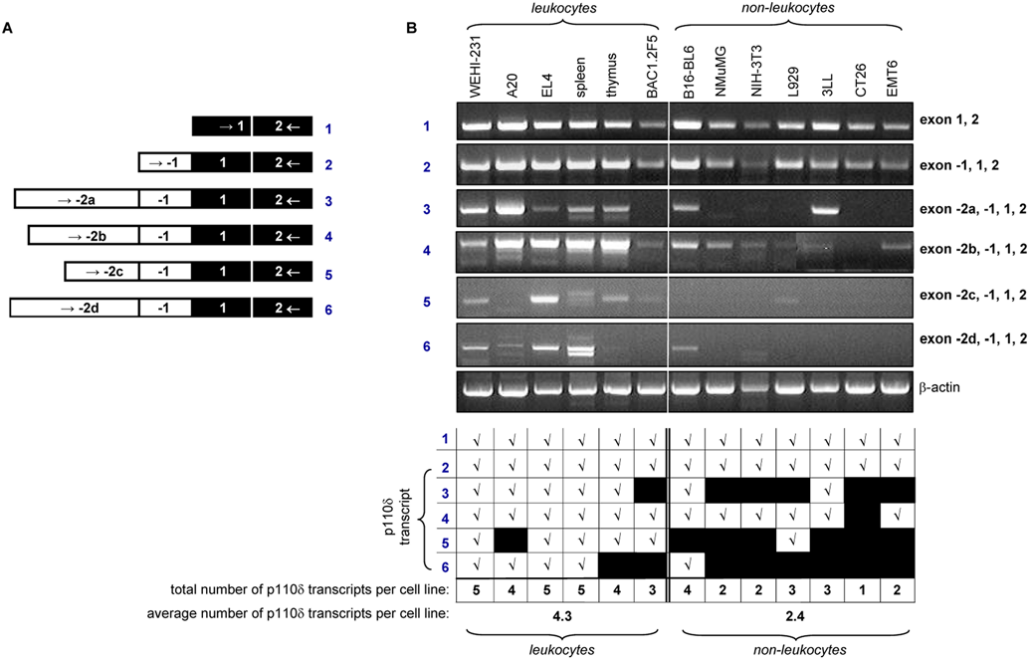
*PIK3CD* transcripts in murine cell lines and tissues, as assessed by RT-PCR (A) Schematic representation of PCR primers used to detect distinct murine *PIK3CD* transcripts. In each case, a reverse primer in exon 2 and a forward primer in exon 1, -1, -2a, -2b, -2c or -2d, was used. (B) Agarose gel analysis of PCR products generated by RT-PCR (40 cycles) using primers for the different p110δ mRNA transcripts in panel of murine cell lines and tissues, with the observations summarized underneath.

We next used RT-PCR to confirm the presence of the different p110δ transcripts identified by 5′RACE and to determine which of these can be found in a panel of murine leukocyte and non-leukocyte cell lines. Forward primers, specific for each of the 5′ untranslated exons were designed, and used in combination with a common reverse primer in exon 2 (schematically shown in Figure 5A). PCR products of the predicted size were purified by agarose gel electrophoresis and verified by DNA sequencing (data not shown).

All cell lines tested (except CT26 colon carcinoma) expressed at least one type of transcript containing a -2 exon (Figure 5B). Leukocytes contained a broader variety of p110δ transcripts than non-leukocytes, with an average number of distinct p110δ transcripts of 4.3 *versus* 2.4 in leukocytes and non-leukocytes, respectively (summarized in bottom panel of Figure 5B).

**Figure 6:**
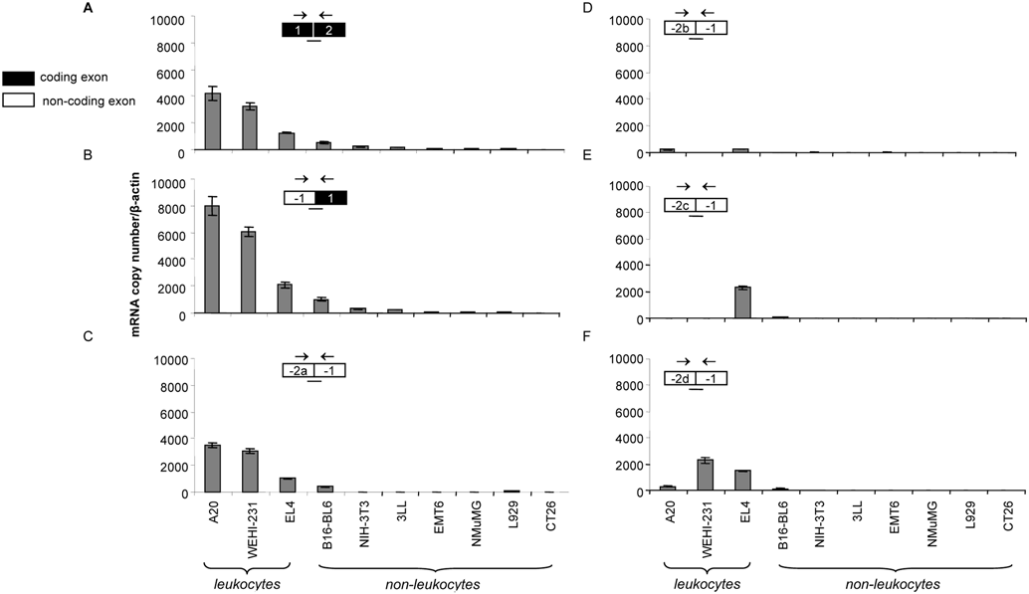
*PIK3CD* transcripts in murine cell lines, as determined by real time PCR. Absolute quantification of the different p110δ transcripts in a panel of murine cell lines by real time RT-PCR (3 experiments) using primer mixes containing a forward primer in the first exon of each transcript, a reverse primer in the subsequent exon and a probe overlaying the exon/exon boundary. Copy numbers were calculated using a standard curve with the different transcripts cloned into a plasmid, and used as a control template for PCR. Samples were normalized to the levels of β-actin mRNA. The different panels represent amplification of the boundaries of (A) exon 1/exon 2; (B) exon -1/exon 1; (C) exon -2a/exon -1 (D) exon -2b/exon -1 (E) exon -2c/exon -1 (F) exon -2d/exon -1.

To more accurately quantify the amount of each p110δ transcript, we next used real time RT-PCR (Figure 6). For each transcript, the PCR reaction consisted of a forward and reverse primer, which were designed to amplify a cDNA sequence of ∼100 bp spanning an exon boundary specific to the transcript, and a dye-emitting probe, which bound at a sequence overlaying this exon boundary. During amplification of the cDNA sequence, cleavage of a reporter dye from the probe results in fluorescence emission, which can be directly correlated with the level of each particular p110δ transcript. For example, to measure the amount of p110δ transcripts containing exon -2a, a PCR was performed using a forward primer in exon -2a and a reverse primer in exon -1, while the dye-emitting probe bound at this exon-exon boundary. The fluorescence detected from this PCR reaction therefore represents the amount of all transcripts specifically containing exons -2a and -1.

These experiments revealed that leukocytes express significantly higher amounts of the different p110δ transcripts than non-leukocytes (Figure 6), indicating that leukocytes are likely to be more efficient at using p110δ gene promoters than non-leukocytes.

In all cell lines, the transcript containing the first coding exon (exon 1; Figure 6A) was expressed at similar levels as the transcript containing the exon -2a/exon -1 boundary, which is the most abundantly expressed -2 exon (Figure 6C).

Surprisingly, the transcripts containing the exon -1/exon 1 boundary (Figure 6B) were two-fold more abundant than the transcripts containing the exon 1/exon 2 boundary (Figure 6A). This indicates that shorter but still fully processed mRNAs (i.e. with a poly A tail since oligo d(T) was used for reverse transcription) are made. These would contain a 5′UTR with at least the untranslated exon -1 and the coding exon 1, but without any of the other coding exons. Database analysis also provided evidence for such shorter p110δ transcripts (marked with asterisks in Figure 4A). These may belong to the recently identified new class of mRNA transcripts that initiate near the expected transcription start sites, upstream of protein encoding sequences [55]–[59].

### *In silico* analysis of *PIK3CD* promoter

**Figure 7:**
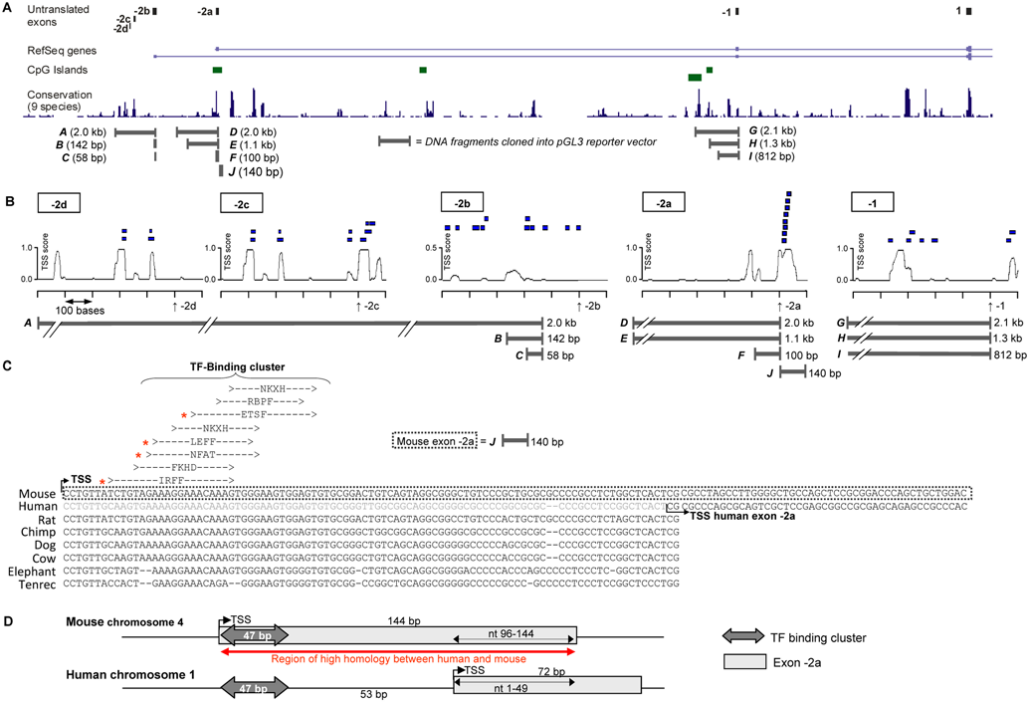
Bioinformatic analysis of potential promoter elements and TF binding sites in *PIK3CD*. (A) Schematic representation of the murine p110δ 5′UTR. The upper panel shows the five untranslated murine p110δ exons (and exon 1) in their genomic context, with below (in descending order): the mouse RefSeq genes, CpG islands, homology with 9 species (rat, human, chimp, rhesus, dog, cow, armadillo, elephant, tenrec, plotted against the murine sequence) and the genomic fragments that were subcloned for use in gene reporter assays. (B) Locations of the conserved TF binding sites in the 600 bases (500 upstream to 100 downstream of the transcript start site) in the forward strand flanking the different 5′untranslated exons of mouse p110δ gene. The exon start sites are indicated by the vertical arrows, the TF binding sites found on the forward strand are shown as blue boxes above the TSS score graphs. Also shown is the degree of cross species (28 species) genomic conservation as calculated by the phastCons program [94] from a minimum of 0.0 to a maximum of 1.0. The genomic DNA fragments subcloned into the PGL3 reporter vector are shown underneath. (C) Alignment and conservation of the TF binding cluster identified in mouse exon -2a with genomic sequences upstream of the translation start site of *PIK3CD* of 7 other species. (D) Schematic representation of TF binding cluster location in relation to exon -2 in human and mouse.

Alignment of the genomic sequence of flanking (and including) the 5′UTR exons of mouse *PIK3CD* with 8 other species revealed high homology in specific areas, indicative for functionally conserved DNA sequences, including 4 CpG islands but no TATA boxes (Figure 7A; Supporting information, File S2).

For each of the murine untranslated exons, the region spanning 500 bp upstream and 100 bp downstream of the first nucleotide were analysed for TF-binding sites and the transcription start site (TSS) prediction score within this region was assessed (Figure 7B). TF-binding sites were identified in the vicinity of all mouse untranslated exons, however a particularly condensed cluster of TF-binding sites was identified *within* exon -2a (Figure 7B, C). Interestingly, in human, this TF-binding cluster lies 5′ of the TSS (Figure 7C; schematically shown in Figure 7D). It is unusual, but not unheard of, that promoter regions are contained *within* exons. Indeed, recent work from the ENCODE project (http://www.genome.gov/10005107 and http://genome.cse.ucsc.edu/ENCODE/) has revealed that proximal TF binding sites usually fall within 1 kb of both sides, 5′ and 3′, of the transcription start site [60].

The TF-binding cluster of murine exon -2a was located within a CpG island (Figure 7A); was associated with a good TSS prediction score (0.9/1.0; Figure 7B) and was highly conserved across 28 species (Supporting information, File S2; Figure 7C shows the high degree homology of this region across 8 species, Figure 7D schematically shows the homology between human and mouse in this area). Collectively, these observations indicate the presence of a putative promoter region in/around exon -2a. Interestingly, 4 of the 7 different TFs identified within this binding cluster, namely ETS, IRF, NFAT and LEF (indicated by an asterisk in Figure 7C), have previously been associated with haematopoiesis and expression of leukocyte-specific genes (discussed in more detail below), suggesting that this TF-binding region may be involved in the high p110δ expression in leukocytes.

### Functional analysis of putative *PIK3CD* promoter elements using reporter assays

We next cloned intronic genomic DNA sequences that flank mouse exons 1, -2a and -2b (including exons -2c and -2d) at their 5′ end (Figure 7A,B; referred to as DNA fragments *A–I*) as well as mouse exon -2a itself (Figure 7B, DNA fragment *J*, which contains the TF-binding cluster), into the pGL3 reporter vector to drive expression of firefly *luciferase*. Vectors were transiently transfected in leukocyte and non-leukocyte cell lines and the promoter activities of the different *PIK3CD* DNA fragments were compared to that of the established leukocyte-specific promoter of Vav [61], and of the SV40 promoter, which is active in all cell types. The pGL3-Basic vector, which does not contain a promoter sequence upstream of firefly *luciferase*, was used to assess the basal level of luminescence.

**Figure 8:**
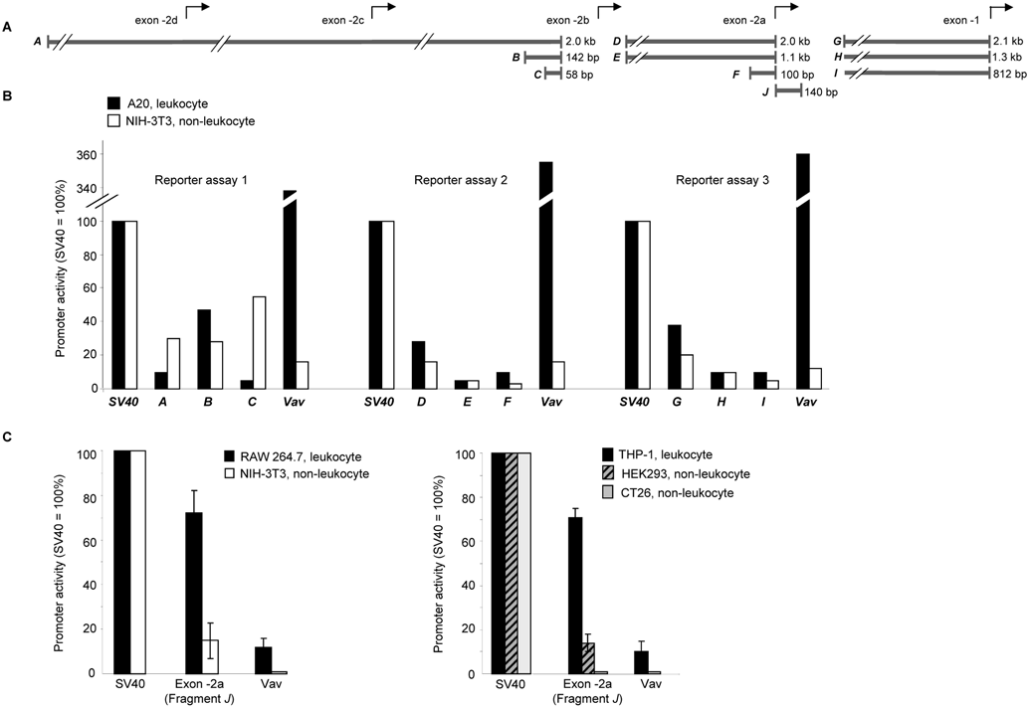
*PIK3CD* promoter analysis by reporter gene assays. (A) Schematic representation of the genomic *PIK3CD* DNA fragments *(A–I)* cloned upstream of firefly *luciferase* in the pGL3 reporter vector. (B) Promoter activity of each of the potential promoter regions *(A–I)* in A20 leukocytes and NIH-3T3 fibroblasts, as determined by luciferase reporter assays. The promoter activity of each *PIK3CD* region and the *Vav* promoter are expressed as a percentage of the SV40 promoter activity after subtraction of basal luminescence. (C) Promoter activity of mouse *PIK3CD* exon -2a DNA in leukocytes (RAW 264.7 and THP-1) *versus* non-leukocyte cell lines (NIH 3T3, HEK 293, and CT26), in two independent experiments. The promoter activity of exon -2a and *Vav* promoter are expressed as a percentage of the SV40 promoter activity after subtraction of basal luminescence. Each transfection was carried out in triplicate with the error bars indicating the standard deviation.

The intronic genomic DNA fragments *A–I* did not possess significantly higher promoter activity in A20 leukocytes compared to NIH 3T3 fibroblasts (Figure 8B). This is in contrast to DNA fragment *J* (mouse exon-2a containing the TF-binding cluster) which had significantly higher promoter activity in the mouse macrophage cell line RAW 264.7 than in NIH 3T3 fibroblasts (Figure 8C), which was significantly higher than the leukocyte-specific *Vav* promoter (Figure 8C). The exon -2 fragment has also higher activity in the THP-1 monocytic cell line compared to the HEK293 (embryonic kidney) and CT26 (colon carcinoma) cell lines, again with higher activity compared to the *Vav* promoter (Figure 8C). Taken together with the relatively high abundance of the -2a transcripts (Figure 6C) over the other p110δ exon -2 transcripts (Figure 6D–F), these data indicate that the TF binding cluster of exon -2a is the predominant promoter of p110δ expression in leukocytes.

## Discussion

In this study, we have explored the mechanisms by which the well-documented leukocyte-enriched expression of p110δ might be achieved. We present evidence that p110δ expression is mainly regulated at the transcriptional level arising from different transcripts. *PIK3CD* transcription appears to be constitutive and not responsive to acute cellular stimuli, at least using the agonists tested in the three cell types (NIH 3T3 and L929 fibroblasts, primary B cells and U937 myelomonocytic cells) used in our study. All cell types can express the distinct p110δ mRNA transcripts but leukocytes express a greater diversity of transcripts and significantly higher amounts of the individual transcripts. In particular, the p110δ transcript containing the untranslated exon -2a, was expressed at similar levels to the transcript containing coding exon 1 in a panel of murine leukocyte cell lines (compare Figure 6A with Figure 6C), indicating that the majority of p110δ transcripts in a cell contain exon -2a.

We have identified a region within mouse exon -2a that contains a cluster of TF-binding sites. This TF-binding cluster is highly conserved between species and found immediately upstream of human exon -2a in human. This TF binding cluster contains at least 4 leukocyte-related TFs sites and was found to display higher promoter activity in leukocyte cell lines compared to non-leukocyte cell lines. Given that the majority of *PIK3CD* transcripts contain exon -2a, these data indicate that the promoter region identified within mouse exon -2a is likely to be sufficient in mediating the majority of leukocyte-specific *PIK3CD* gene expression.

4 of the 7 TFs identified within this binding cluster, namely ETS, IRF, NFAT and LEF have previously been associated with regulation of haematopoiesis and expression of leukocyte-specific genes. Indeed, the ETS family of TFs play important roles in the regulation of haematopoiesis [62]–[68]. IRF family members are highly expressed, but not exclusively, in cells of the immune system and play a pivotal role in the induction of type I IFN signalling pathways [69], proinflammatory cytokines and expression of macrophage and B cell specific genes [69], [70]. NFAT family proteins are also mainly found in cells of the immune system, such as T cells, mast cells, NK cells and monocytes [71] and play a role in the regulation of various cytokines [72]. Finally, the LEF family of TFs, which are highly expressed in pre-B and T lymphocytes [73], have been linked to the regulation and expression of a number of lymphoid-specific genes [74]–[79].

High expression levels of p110δ are also frequently observed in some non-leukocyte cancer cell lines, such as in breast carcinoma, melanoma and glioma [18]. It is possible that cancer cells upregulate or aberrantly express TFs which are, in non-cancer cells, more specific for leukocytes. It is of interest to note that a number of the TFs that bind in the exon -2a cluster have indeed been implicated in breast cancer progression, including LEF [80]–[83], ETS-1 [84], [85], ETS-2 [86] and NFAT3 [87]. Recently, all four of these leukocyte-associated TF were identified as the most frequently differentially activated TFs in breast cancer based on a large microarray dataset [88].

We have found evidence that, among the multiple p110δ transcripts, there may be mRNAs that do not encode full length p110δ. Indeed, transcripts containing the exon -1/exon 1 boundary are more abundant than those covering the exon 1/exon 2 boundary (compare Figure 6B to Figure 6A). Current database information supports the presence of such shorter p110δ transcripts (marked with an asterisk in Figure 4A). Indeed, several recent studies have reported the discovery of a new class of short promoter-associated RNA transcripts that initiate near the expected transcription start sites upstream of protein-encoding sequences [56]–[58] (reviewed in [55]). It remains to be seen whether these RNAs have a function, but their prevalence suggests that their synthesis may serve a functional role.

Further work is required to understand the precise mechanism of p110δ gene expression. The complexity of gene regulation has been exemplified by examination of 400 protein-coding genes in 1% (30 million bases) of the human genome as part of the ENCODE project [60], which revealed that 80% of these genes had additional exons, many of which were located thousands of bases away from the coding exons. Also many novel transcription start sites were found, many located thousands of bases away from the known start sites, while 25% of the promoters discovered were at the 3′ end of the genes rather then at the 5′ end. It is therefore highly likely that p110δ expression will be subject to additional levels of control rather than by simple proximal promoter elements.

The data presented are the first to shed light onto the leukocyte-enriched expression of *PI3KCD*. Further investigations are needed to identify which TF-binding sites are critical in driving *PIK3CD* gene expression and whether cells of non-leukocyte origin, such as breast cancer cells, are able to utilize this putative promoter. Interference with *PIK3CD* expression at the promoter level may offer a novel therapeutic target in cases of aberrant p110δ overexpression, as observed in some cancers [18].

## Materials and Methods

### Antibodies and reagents

Antibodies to class IA PI3Ks were generated in-house or purchased from Santa Cruz Biotechnology (p110β, sc-602). Cell culture reagents were purchased from Invitrogen, recombinant mouse TNF was provided by Peter Brouckaert (Ghent University, Belgium), other reagents were from Sigma: Actinomycin D (856258), 5′-azacytidine (A2358), trichostatin A (T8552), antibodies to β-actin (A5441).

### RNA extraction, 5′Rapid Amplification of cDNA Ends (5′RACE), Reverse Transcription (RT)-Polymerase Chain Reaction (PCR) and Real Time RT-PCR

Total RNA was extracted from cells using the RNeasy mini kit (Qiagen, 74104). mRNA was subsequently reverse transcribed using SuperScript II Reverse Transcriptase (Invitrogen, 18064) and oligo d(T) primers, and subsequently used in 5′RACE, RT-PCR or real time PCR.

For 5′RACE, the FirstChoice® RLM-RACE kit (Ambion, 1700) was used following the manufacturer's protocol using the following outer primers: murine 5′-CAGATCAGCTCCTCATTGGCACT-3′, human 5′-GCTTCTTCACGCGGTCGCCC-3′ and inner primers: murine 5′-ACTTGAACTTCCCCGTGTCCCG-3′, human 5′-CGGGACACAGGGAAGTTCAGGT-3′. Products were cloned into pGEM-Teasy (Promega) for sequencing. RT-PCR for mouse p110δ was carried out using a common reverse primer in exon 2 (5′-TGCCAATGAGGAGGCTGATCTG-3′) in combination with exon-specific forward primers, as follows: for exon 1: 5′-CGTGGTTGTTGACTTCTTGC-3′; for exon -1: 5′-GAGAGCCAGGCAGAAGTGGGAT-3′; for exon -2a: 5′-GAAGTGGAGTGTGCGGACTGTC-3′; for exon -2b: 5′-GCATCAACTCCTGCCCTGTGTG-3′; for exon -2c: 5′-GCCATGCTATCGGGAACTTGAG-3′; for exon -2d: 5′-CAGAGTGCTTCCGGTGGTATCC-3′. For RT-PCR of human p110δ, the following primers were used: common reverse primer in exon 1: 5′-CGGGACACAGGGAAGTTCAGGT-3′ in combination with the following exon-specific primers: for exon -1: 5′-TAAGGAGTCAGGCCAGGGCGG-3′, for exon -2a: 5′-AGTCGCTCCGAGCGGCCGCG-3′, for exon-2b: 5′-CGAGGTTGGGAGAGGAGTGTG-3′. RT-PCR products were cloned into pGEM-Teasy vector (Promega) and sequenced using the T7 primer.

For real time RT-PCR amplification TaqMan Universal PCR Mastermix (4304437) and primer mixes containing a FAM reporter probe (TagMan Gene Expression Assay) were obtained from Applied Biosystems. SYBR Green (Qiagen, 204143) was used for quantifying 18S RNA. Exon-specific primer sets and probes were designed to identify transcripts containing exon -1, -2a, -2c and -2d. For exon -2a the following primer sequences were used; forward primer 5′-TCGCGCCTAGCCTTGG-3′, reverse primer 5′-GGCATCAGCGGGCTTCA-3′ and FAM reporter sequence 5′CTCAGCTCCTTAGATGTCGGTC-3′. For exon -2b the following primer sequences were used; forward primer 5′-AGTGTCTGTCCTGACTTCCTAAGAA-3′, reverse primer 5′-CGGGCTTCATCCCACTTCTG-3′ and FAM reporter sequence 5′- CAGCTCCTTAGATGTACTTCTACA-3′. For each transcript of interest, known amounts of plasmids with this transcript were used to create a standard curve. Real-time PCR generated a series of C_T_ values (the PCR cycle at which amplification of each target gene is first detected) for endogenous and plasmid-born cDNA, which allowed for the determination of mRNA copy numbers for each individual gene.

### Western blot

Cells were lysed and immunoblotted for PI3K expression as described before [89]. Primary antibodies were detected using fluorescently-labeled species-specific secondary antibodies (anti-mouse IRDye 800-conjugated (Rockland) and anti-rabbit Alexa-Fluor 680-conjugated (Molecular Probes). Quantification was done using an Odyssey infrared scanner (LICOR) using the manufacturer's software. Signal intensities were normalized for an internal loading control such as β-actin or GAPDH.

### Bioinformatic analysis of putative promoter elements in *PIK3CD* genes

The upstream sequences for the five untranslated exons of the murine p110δ gene were inspected within the February 2006 (NCBI build 36) assembly of the Mouse genome using the UCSC genome browser [90]. Regions spanning 500 bp upstream and 100 bp downstream of the first nucleotide of each exon were analysed. The corresponding multiple species alignment was extracted using the Vertebrate Multiz Alignment & Conservation track [91] within the UCSC genome browser. The alignments were then screened for conserved TF binding sites using MatInspector [92] and a vertebrate factors subset of a of a proprietary database of Genomatix. In addition the candidate regions were inspected with Eponine [93], a probabilistic method for detecting transcription start sites, using a threshold of 0.9.

### Reporter gene assays

PCR amplification of genomic DNA from C57Bl/6 mice was used to generate fragments for the reporter assays. The amplified PCR products were inserted into the pGL3 reporter vector (Promega). Transfections of NIH3T3 and A20 cells were performed using Qiagen Superfect or electroporation, respectively. Equal number of cells were washed and lysed, using Promega lysis buffer (to normalize for transfection efficiency) and then assayed for luciferase activity using the firefly luciferase substrate from Promega on the MicroBeta workstation (Perkin Elmer). The luciferase activity was normalized using a luciferase gene in a pGL3 reporter vector under the control of the SV40 promoter as well as a promoterless luciferase/pGL3 reporter vector. DNA of the lymphocyte-specific Vav promoter (construct HS21 mentioned in Ref. [61], which we cloned from the original β-galactosidase reporter construct into a pGL2 luciferase reporter vector) was used as a positive control.

## Supporting Information

File S1 5′ RACE product sequences Sequences of the different murine (Mm) and human (Hs) p110δ transcripts as identified by 5′RACE.
(0.03 MB DOC)

File S2 Annotated multiple species alignments. This file shows the conserved TF binding sites for the different murine and human p110δ mRNA transcripts as identified by 5′ RACE.
(0.19 MB DOC)
