# Supplementary material for: Regulation of p110δ PI 3-Kinase Gene Expression
Source: PLoS One. 2009 Apr 9;4(4):e5145. doi: 10.1371/journal.pone.0005145 (PMC2663053; doi:10.1371/journal.pone.0005145)
Supplement: File S1 — 5′ RACE product sequences Sequences of the different murine (Mm) and human (Hs) p110δ transcripts as identified by 5′RACE. (0.03 MB DOC) [file pone.0005145.s001.doc]

**Additional file 1 - 5’ RACE product sequences**

Sequences of the different murine (Mm) and human (Hs) p110δ transcripts as identified by 5’RACE. Exons -2a, -2b (human and mouse), -2c and -2d (mouse) in **bold**, exon -1 underlined and exon 1 in normal font with the ATG translation start site in **bold and green** and the in-frame upstream TAA stop codon in bold and red.

**5’ RACE product sequences**

> Mm_PIK3CD, **exon -2d**, exon –1 and exon 1:

**TAGGACTTCTTTCCTCTCCCATCAAATGGGGCCAGAGTGCTTCCGGTGGTATCCACCTCCAGAGAGAAGGACCCACGTGAGCTCCGAGGGTGGCTTTGCTATGGCAAGTCCTTTTTGGGTTGCTTCAGATGACCCGCCATGAGCAGTTCG**ACATCTAAGGAGCTGAGAGCCAGGCAGAAGTGGGATGAAGCCCGCTGATGCCAAAGTACCTTTAATCTCCCAGGCAGAGGGGCCTTGGCTGGTGGTCCTTCTTGGCCCATACCAAACAGGAAAACAGACCTCAGAGAAG**TAA**CACAACAGG**ATG**CCCCCTGGGGTGGACTGCCCCATGGAGTTCTGGACCAAAGAGGAGAGCCAGAGCGTGGTTGTTGACTTCTTGCTGCCCACAGGGGTCTACTTGAACTTCCCCGTGTCCCGCAATGCCAACCTCAGCACCATCAAGCAG

> Mm_PIK3CD, **exon -2c**, exon –1 and exon 1:

**ATCAGAAACCAGAGAGGGGAAGAAGGCCGGAGGCCATGCTATCGGGAACTTGAGGAATGAGGACGGCTCTACTCAAAT**ACATCTAAGGAGCTGAGAGCCAGGCAGAAGTGGGATGAAGCCCGCTGATGCCAAAGTACCTTTAATCTCCCAGGCAGAGGGGCCTTGGCTGGTGGTCCTTCTTGGCCCATACCAAACAGGAAAACAGACCTCAGAGAAG**TAA**CACAACAGG**ATG**CCCCCTGGGGTGGACTGCCCCATGGAGTTCTGGACCAAAGAGGAGAGCCAGAGCGTGGTTGTTGACTTCTTGCTGCCCACAGGGGTCTACTTGAACTTCCCCGTGTCCCGCAATGCCAACCTCAGCACCATCAAGCAG

> Mm_PIK3CD, **exon -2b**, exon –1 and exon 1:

**AAGAAAGCAGGCCCAGCCAGCCAGTCAGCAGCATTCCTCCGAGGTCTCTGCATCAACTCCTGCCCTGTGTGAGTGTCTGTCCTGACTTCCTAAGAAGACGAACAGTGATGTAGAAGT**ACATCTAAGGAGCTGAGAGCCAGGCAGAAGTGGGATGAAGCCCGCTGATGCCAAAGTACCTTTAATCTCCCAGGCAGAGGGGCCTTGGCTGGTGGTCCTTCTTGGCCCATACCAAACAGGAAAACAGACCTCAGAGAAG**TAA**CACAACAGG**ATG**CCCCCTGGGGTGGACTGCCCCATGGAGTTCTGGACCAAAGAGGAGAGCCAGAGCGTGGTTGTTGACTTCTTGCTGCCCACAGGGGTCTACTTGAACTTCCCCGTGTCCCGCAATGCCAACCTCAGCACCATCAAGCAG

>Mm_PIK3CD, **exon -2a**, exon –1 and exon 1

**CCTGTTATCTGTAGAAAGGAAACAAAGTGGGAAGTGGAGTGTGCGGACTGTCAGTAGGCGGGCTGTCCCGCTGCGCGCCCCGCCTCTGGCTCACTCGCGCCTAGCCTTGGGGCTGCCAGCTCCGCCGACCCAGCTGCTGGACCG**ACATCTAAGGAGCTGAGAGCCAGGCAGAAGTGGGATGAAGCCCGCTGATGCCAAAGTACCTTTAATCTCCCAGGCAGAGGGGCCTTGGCTGGTGGTCCTTCTTGGCCCATACCAAACAGGAAAACAGACCTCAGAGAAG**TAA**CACAACAGG**ATG**CCCCCTGGGGTGGACTGCCCCATGGAGTTCTGGACCAAAGAGGAGAGCCAGAGCGTGGTTGTTGACTTCTTGCTGCCCACAGGGGTCTACTTGAACTTCCCCGTGTCCCGCAATGCCAACCTCAGCACCATCAAGCAG

> Mm_PIK3CD, exon –1 and exon 1

ACATCTAAGGAGCTGAGAGCCAGGCAGAAGTGGGATGAAGCCCGCTGATGCCAAAGTACCTTTAATCTCCCAGGCAGAGGGGCCTTGGCTGGTGGTCCTTCTTGGCCCATACCAAACAGGAAAACAGACCTCAGAGAAG**TAA**CACAACAGG**ATG**CCCCCTGGGGTGGACTGCCCCATGGAGTTCTGGACCAAAGAGGAGAGCCAGAGCGTGGTTGTTGACTTCTTGCTGCCCACAGGGGTCTACTTGAACTTCCCCGTGTCCCGCAATGCCAACCTCAGCACCATCAAGCAG

> Hs_PIK3CD, **exon -2b**, exon –1 and exon 1

**GAGGCGCCCAGGACACCACGAGGTTGGGAGAGGAGTGTGCTTTGCACTCTGCACTCTCCAGCTCAGAGTAGCTGAGGATGCTAGGGAGACTGGGGGCTGGCCCACGGGGGGAGCAGAGGAGTCCTCAGTGCGGTCCTCACTCTGAAAACAGCATTTTTCCCTAGGCTTGAGAATGCCTCCGGGATTGGACTGATCCAGGCTGGACCCACGTCTGTCTGGTGATACCAGGGGCAGAGGGACCACTCTGACAG**ATAAGGAGTCAGGCCAGGGCGGGATGACACTCATTGATTCTAAAGCATCTTTAATCTGCCAGGCGGAGGGGGCTTTGCTGGTCTTTCTTGGACTATTCCAGAGAGGACAACTGTCATCTGGGAAG**TAA**CAACGCAGG**ATG**CCCCCTGGGGTGGACTGCCCCATGGAATTCTGGACCAAGGAGGAGAATCAGAGCGTTGTGGTTGACTTCCTGCTGCCCACAGGGGTCTACCTGAACTTCCCTGTGTCCCGCAATGCCAACCTCAGCACCATCAAGCA

> Hs_PIK3CD, **exon -2a**, exon –1 and exon 1

**AGTCGCTCCGAGCGGCCGCGAGCAGAGCCGCCCAGCCCTGCCAGCTGCGCCGGGAC**ATAAGGAGTCAGGCCAGGGCGGGATGACACTCATTGATTCTAAAGCATCTTTAATCTGCCAGGCGGAGGGGGCTTTGCTGGTCTTTCTTGGACTATTCCAGAGAGGACAACTGTCATCTGGGAAG**TAA**CAACGCAGG**ATG**CCCCCTGGGGTGGACTGCCCCATGGAATTCTGGACCAAGGAGGAGAATCAGAGCGTTGTGGTTGACTTCCTGCTGCCCACAGGGGTCTACCTGAACTTCCCTGTGTCCCGCAATGCCAACCTCAGCACCATCAAGCA

> Hs_PIK3CD, exon –1 and exon 1

ATAAGGAGTCAGGCCAGGGCGGGATGACACTCATTGATTCTAAAGCATCTTTAATCTGCCAGGCGGAGGGGGCTTTGCTGGTCTTTCTTGGACTATTCCAGAGAGGACAACTGTCATCTGGGAAG**TAA**CAACGCAGG**ATG**CCCCCTGGGGTGGACTGCCCCATGGAATTCTGGACCAAGGAGGAGAATCAGAGCGTTGTGGTTGACTTCCTGCTGCCCACAGGGGTCTACCTGAACTTCCCTGTGTCCCGCAATGCCAACCTCAGCACCATCAAGCA
