# Supplementary material for: Regulation of p110δ PI 3-Kinase Gene Expression
Source: PLoS One. 2009 Apr 9;4(4):e5145. doi: 10.1371/journal.pone.0005145 (PMC2663053; doi:10.1371/journal.pone.0005145)
Supplement: File S2 — Annotated multiple species alignments. This file shows the conserved TF binding sites for the different murine and human p110δ mRNA transcripts as identified by 5′ RACE. (0.19 MB DOC) [file pone.0005145.s002.doc]

**Additional File 2**

**Annotated multiple species alignments**

For each observed transcript, the 500 bp upstream to 100 bp downstream of their observed transcription start was used to extract a multiple species alignment from the UCSC genome browser, which was then refined to remove non-mouse insertions. The alignments were then screened for conserved transcription factor binding sites using MatInspector and a vertebrate factors subset of Genomatix's proprietary database. In addition the candidate regions were inspected with Eponine, a probabilistic method for detecting transcription start sites, using a threshold of 0.9, or a threshold of 0.5 if none were predicted.

Transcription factor binding sites that were found to be present, at the same positions, in at least 2/3 of the species (including mouse or rat) are marked onto the alignment as arrows. The arrows are above the blocks of the alignment, pointing left to right for the forwards strand, and below the blocks of alignment, pointing right to left for the reverse strand. A list of the transcription factors found, and their descriptions, is given after each alignment.

The intronic sequence is in small letters, the first 100 nucleotides of the first exons of each transcript are in capital letters.

**Annotated genomic alignment -500 to +100 of Mouse exon -1**

Mouse cagctgaccttctcatctctaacccagcagttgaattttacttcgacttggctttgggga

Rat cagctgagcttctcatctctaacacaacaggtgaatctccctttgccttggctctggaaa

Human cactcttcctttttatctccagcccaatacatgaattttcctttgacctgggccttgaaa

Chimp cactcttcctttttatctccagcccaatacatgaattttcctttgacctgggcctcgaaa

Rhesus cactcttcctttttatctccagcccaatagatggattttcctttgacctgggccttgaaa

Dog cacccatccttttcatctccagca-agcagatgagtctccctgtaatttagtacttga--

Cow caccattccctttcatctccacaa-agcagataaatcctcctttgatctgggtgttga--

Armadillo cgccctttgttttcatttccagc---------------tccttcaaattggaccttgaag

<---GATA----<

Mouse atgaaggtctgaagtgcacgaggatctgagcctaaatctgaattccattctccttttacc

Rat ----------gagctacatggggaaatga----agacctaaagtgcatgaacttttattt

Human aca-------gatccacatgggga-ataa----agatttcaagttcaccaacctgct--t

Chimp aca-------gatccacatgggga-ataa----agatttcaagttcaccaacctgct--t

Rhesus aca-------gatctacatgggga-ataa----agatttcaagttcaccaacctgca--t

Dog -------------------------gtag----ggacctcaagtgcaggaatctgct--t

Cow -------------------------atag----agacctcaagttcacagacatgtt--t

Armadillo aca-------gaaactgttaggga-atcc----tgatcccaagttcacctgcctgct--t

>-----HOXF------>

Mouse ttttcagccacgcaatggaattcaaacttaggcatggctccgatcaactaactgtactta

Rat cttgaagccacgtaatgggattcgaacttatgcagggctcagatcaattaactctgctta

Human tttgcatccatcgaatggaactcgaacccaggcttggctcagatcaatta-ctttgctca

Chimp tttgcatccatcgaatggaactcgaacccaggcttggctcagatcaatta-ctttgctca

Rhesus tttgcacccatcgaatggaactcgaacccaggcttggctcagatcaatta-ctttgctca

Dog tttgcatccattgaatggaacttgaacccaggcttgacaca-------------------

Cow tttgcagccattgaatggaactcgaacccaggcttgactcagatcactta-ctttgctca

Armadillo ttcccagcccatgaatgggactggaacccaggcttggctcagatcactta-ctttgctca

<---GATA----<

<-----HOXF------<

<------CLOX-------<

<----NKXH-----<

>-------PTF1-------->

>-----TBPF------>

Mouse aaaaaccaaccagcagcaataaaagaccatctgtgtatacccttgcgtcaggtctgggga

Rat cgaaaccaaccagcagctctaaacgaccatctgtgtgcatcccaccttcaggtctgggaa

Human aaaaaccaacaaacagctctaaaa-atcatctgtgcacattcctgctccaggcctggaaa

Chimp aaaaaccaacaaacagctctaaaa-atcatctgtgcacattcctgctccaggcctggaaa

Rhesus aaaaaccaacaaacagctctaaaa-atcatctgtgcacattcctgctccaggcctggaaa

Dog ----accaatgaataactctaaaa-atcatct-tatga--tcttgcttcaggtctggaaa

Cow aataaccaacaaacaactctagaa-atcatctgtataaattcctgcttcggtcctggaaa

Armadillo aaaaaccggcagacagctctaaaa-atcatc--tgtgtattcctgcctcaggcctgg-aa

<-----AP4R------< <-----PAX6---

>----HAML----> >-----CREB---

Mouse tacccctgtggtgtatactacttctatgctaccaacgggaactgggctggggttgacaca

Rat tgaccctgtgct-----ttacttctgtgctggcaaagtggactggggtggggttgacaca

Human tgctcctgtggt-----tggtttcagtaccaccaaagggagctggggtggcattgacgca

Chimp tgctcctgtggt-----tggtttcagtaccaccaaagggagctggggtggcattgacgca

Rhesus tgctcctgtggt-----tggtttcagtaccaccaaagggagctggggtggcattgacgca

Dog tgctccgatggt-----ttgcttcagtgccatcaaaggg-gctggggtggcattgacaca

Cow tgttcctgtggt-----ttgcttcagtaccaccaaagagagctggggtggcattgacaca

Armadillo cgctcctgtgtt-----ttgcctcagtaccaccaaagggagctggggtggcggtgatgca

-----< <--------ZBPF---------<

<---------AP1R--

--->

Mouse ggtatcgagaaaggcctacccagcaaagtggggttgtcttacaagaagggaatctgagat

Rat ggtagctagataggttgacccagcaaattgggcttggcttacaagaaggggatctgagat

Human gatggcaagaaaggccgatcaagtggaatgtgattggtttgcaaaaaggagagcctagat

Chimp gatggcaagaaaggccgatcaagtggaatgtgattggtttgcaaaaaggagagcctagat

Rhesus gatggcaagaaaggccgatcaagtggaatgtgattggtttgcaaaaaggagagcggagat

Dog ggtggtgggaaaggcccatcaagcagaatgtgattggtttgcaaaatgggag-cctaggt

Cow gatggtgaaaaaggcccgtcaagcacaatgtgactggtttgcaaaaagggaa-ccttggt

Armadillo gatggcgagaaaggcccgtgaagcagaatgtgactcgtttgcaaaaagggaa-acgaggt

--------<

Mouse gaaatggcccttccttcagtgctgggggctttttgcatgttaggcaggcacggccactga

Rat gaaatggggcttccttcagtactgggagctctttgcgtgttgggcaggca--aacact--

Human gaagcag-------------------------ttg-attttgagctaac-----------

Chimp gaagcag-------------------------ttg-attttgagctaat-----------

Rhesus gaggcag-------------------------ttg-attttgagctaac-----------

Dog gaaacag-------------------------ttg-ctttcaagctaaaacggtttgtga

Cow gaaagag-------------------------ttg-cttttgagctgaagtggtttctga

Armadillo gaaacgg-------------------------tag-tgtttgagct--------------

Mouse gccctgagatatatcatcagcatgtactggattctgaaattctgtgtttaaagcgttctt

Rat gccctgagctacatggtcagcat------gggtctgaacgtctgtgttcaaagtgttctc

Human gtcctgtaaga--------gaatgcacaggtttctgaaattctttgttaaaagttttctt

Chimp gtcctgtaaga--------gaatgcacaggtttctgaaattctttgttaaaagttttctt

Rhesus gtcctataaga--------gaatgcacaggtttctgaaattctttgttaaaagttttctt

Dog gactt-----------------------------taaaaatgtccgttcaaatcattctt

Cow gactt-------------------------------------------------------

Armadillo -----gtaaga--------gaatgtaaaggtttctgaggttct-----------------

|--------------------------- transcript1

|----------------------------------- ESTs

Mouse tcctctttgctttctttcagACATCTAAGGAGCTGAGAGCCAGGCAGAAGTGGGATGAAG

Rat tcctcctttcttcctttcagACACATAAGGAGCTGAGAGCCAGGCAGAAGTGGGATGAAG

Human tctttctttctttctttccaACAGATAAGGAG-------TCAGGCCAGGGCGGGATGACA

Chimp tctttctttctttctttccaACAGATAAGGAG-------TCAGGCCAGGACGGGATGACA

Rhesus tctttcttttcttttttccaACAGATGAGGAG-------TCGGGCCAGGGCGGGATGACA

Dog tctttctttctttttttcagACAGATAAGGAGCTCAGAGTCAGGCAGGGGCGGAATGGTG

Cow ------ttgctctctttcagACAGATAAGGAGCTCAGAGTCAGGCAAGAGTGCATTGATG

Armadillo ---ctctctcttttcttcagACAGATAAGTTGCTGAGGGTGAGCCC--GGCGGAATGA--

tss

> >-------MOKF-------->

>-----HOXF------> <TS> <-TSS->

Mouse CCCGCTGATGCCAAAGTACCTTTAATCTCCCAGGCAGAGGGGCCTTGGCTGGTGGTCCTT

Rat CTCCCTGATGGCAAAGCACCTGTAATCTCCCAGGCACAGGGGCCTTGGCTGGTGGTCCTT

Human CTCATTGATTCTAAAGCATCTTTAATCTGCCAGGCGGAGGGGGCTTTGCTGG---TCTTT

Chimp CTCATTGATTCTAAAGCATCTTTAATCTGCCAGGCGGAGGGGGCTTTGCTGG---TCTTT

Rhesus CTCATTGATTCTAAAGCATCTTTAATCTGCCAGGCGGAGGGGGCTTTGTTGG---TCTTT

Dog CTCATTGATTCCAAAGCATCTCTGATCTGCCAGACAGAGGGGCCTTTGCTGCTTTTCTTT

Cow CTCATTGATTCCAAAGCATCTTTAACCTGTCAGGCAGAGGGACCTTTGCTGCTGGTTTTT

Armadillo -------------AGGCATCTTTAATCTGCCAGGCAGAGGAGCCTTTGCTGGTGGCTTTT

AP1R - MAF and AP1 related factors

AP4R - AP4 and related proteins

CLOX - CLOX and CLOX homology (CDP) factors

CREB - Camp-responsive element binding proteins

GATA - GATA binding factors

HAML - Human acute myelogenous leukemia factor

HOXF - Factors with moderate activity to homeo domain consensus sequence

MOKF - Mouse Krueppel like factor

NKXH - NKX homeodomain factors

PAX6 - PAX-4/PAX-6 paired domain binding sites

PTF1 - Pancreas transcription factor 1, heterotrimeric transcription factor

TBPF - Tata-binding protein factor

ZBPF - Zinc binding protein factors

TSS - predicted Transcription start sites ≥ 0.9 (none)

tss - predicted Transcription start sites ≥ 0.5

**Annotated genomic alignment -500 to +100 of Mouse exon -2a**

Mouse accactagatattcttgtttgtttgtttggtttttgtttttttttgagacagggtttctc

Rat aacactggatgttttt----------------------tattttcaagatggggtctctt

Human ------------------------------------------------------------

Chimp ------------------------------------------------------------

Dog ------------------------------------------------------------

Cow ------------------------------------------------------------

Elephant ------------------------------------------------------------

Tenrec ------------------------------------------------------------

Mouse tgtgtagccctggctgtcctagaactcactctgtagaccaggctggctttgaactcagag

Rat t----agccctagctgtcctggaactccactgctacaccaggttggctttaaac--agag

Human ------------------------------------------------------------

Chimp ------------------------------------------------------------

Dog ------------------------------------------------------------

Cow ------------------------------------------------------------

Elephant ------------------------------------------------------------

Tenrec ------------------------------------------------------------

Mouse acctacgagcctctgcccaagtgctgagattcaaagctagagcaaccatgcctggctaat

Rat acccatgtgcctctgcccaagtgctgagattcaaagcaagggcaaccatgcctagctgat

Human ------------------------------------------------------------

Chimp ------------------------------------------------------------

Dog ------------------------------------------------------------

Cow ------------------------------------------------------------

Elephant ------------------------------------------------------------

Tenrec ------------------------------------------------------------

Mouse gtttttaaaggacctttgagacttgtaggctgtctctgtaaatcttctacactggcctct

Rat gtt-ttaaaggacctttgagacttggggactgcctctgtcactcctctgaattggctgct

Human ------------------------------------------------------------

Chimp ------------------------------------------------------------

Dog ------------------------------------------------gcact----gcc

Cow ------------------------------------------------------------

Elephant ------------------------------------------------------------

Tenrec ------------------------------------------------------------

Mouse agagctcctaagacagtagccactcaccctgtagtactcttagcttgtctaggttttggt

Rat agagcgctcaggactgcagccacccctcctgtcgtactcccagacggtctaggttttgtt

Human --ggtgcctagagctgcagcccctcttcttctcctgctctcag----------cctttct

Chimp -------------------------------------------ctgctttcagcctttct

Dog cgggtgcctagaactgcagccaccctccctctcccgccctcca-----------ctttct

Cow ------------------------------------------------------------

Elephant ------------------------------------------------------------

Tenrec ------------------------------------------------------------

Mouse tttgctttgttgttgttttctgagtgactgtggcgatgctcagggctctccagaccttta

Rat tttgctttgttgttgttttctgagtgactgtggcggtgcctagggctctccggaccttta

Human cccgctctgctgttctttcctgcgtgaaggcggagcgcttaaaggcgcctaggaccttta

Chimp cccgctctgttgttctttcctgcgtgaaggcggagcgcttaaaggcgcctaggaccttta

Dog ctggccatgtggttcttgg-tgtgtgaacgtggagggttttatgggctttaggaccttta

Cow ------------------------------------------------------------

Elephant ------------------------------------------------------------

Tenrec -------------------------------------gtcctgggctctcggggtgtgta

>-

Mouse tttggcagtgtgcctttgcatcaagggaagactttgtttttgaacagagctggggttcga

Rat tttggccatgggc-tttgcatcaagagaagactttgtttttgaacagtgctggggttcaa

Human tttcgttgttttcctttgcataaaaagaaaactt-gttttgtagaagggcgcagcttcca

Chimp tttcgttgttttcctttgcataaaaagaaaactt-gttttgtagaagggcgcagcttcca

Dog tttggttgtttttctttgtataaaaagaaaactt-gttttgaagaagggcgcagcttcca

Cow ------------cttctggatacagataaaactt-gttttgaagaagggcgtagcttcca

Elephant ------------------------------------------------------------

Tenrec gttggttgttttccttcgtataaaaagaaaactt-gttttgaaggcgggc----------

<-

-----MYOD------> >----SP1F----->

Mouse aggcacctgctggtgctaccctcgcagggcccgctgggcttggggcggggctggagaggc

Rat aggcacctgc-ggcgctactcttgcagggcccgctgggcttggggcggggctggagaggc

Human aggcacctggtgatgcccctctagcggggcgaactggggagtgtgggcggcgggggaagc

Chimp aggcacctggtgatgcccctctagcggggcgaactggggggtgtgggcggcgggggaagc

Dog a--cacctggtgctgctcctccagcggggggtggagaggagggtggggggtggggggggg

Cow aggcacctggtgacgcccctccagcgggggcaggcgaggcaggcggagggttagaagaag

Elephant ---cacctgc-gatacccttccatcag-------------ggcctgggggcgggaccaga

Tenrec ---cacctgc-gatgcccctccagcag-------------ggg-tgggggcggg-cgaag

---MYOD------< <--------ZBPF---------<

<--------ZBPF---------

>----NKXH----->

>----RBPF----->

>-------ETSF-------

>----NKXH----->

>-----LEFF------>

<TSS-> >------NFAT------->

>-----FKHD------>

>-------IRFF-------->

<TSS-> |---------------- RefSeq / ESTs

|-------------------- transcript2 / ESTs

Mouse gtagaggatcagttgtttttCCTGTTATCTGTAGAAAGGAAACAAAGTGGGAAGTGGAGT

Rat gtaggggaccagttgtttttCCTGTTATCTGTAGAAAGGAAACAAAGTGGGAAGTGGAGT

Human ctggaggacctgttgtttttCCTGTTGCAAGTGAAAAGGAAACAAAGTGGGAAGTGGAGT

Chimp ctgggggacctgttgtttttCCTGTTGCAAGTGAAAAGGAAACAAAGTGGGAAGTGGAGT

Dog caggaggacctgttgtttttCCTGTTGCAAGTAAAAAGGAAACAAAGTGGGAAGTGGAGT

Cow caggaggacctgctgtttttCCTGTTGCAAGTAAAAGGGAAACAAAGTGGGAAGTGGAGT

Elephant caagagggccggttcttttcCCTGTTGCTAGT--AAAAGAAACAAAGTGGGAAGTGGGGT

Tenrec gtagagga---gctgtttttCCTGTTACCACT--GAAGGAAACAGA--GGGAAGTGGGGT

< <--------ETSF---------<

<--CIZF---<

-> TSS

<-> <--TSS--> |-- ESTs

|------------------------------------------ ESTs

Mouse GTGCGGACTGTCAGTAGGCGGGCTGTCCCGCTGCGCGCCCCGCCTCTGGCTCACTCGCGC

Rat GTGCGGACTGTCAGTAGGCGGCCTGTCCCACTGCTCGCCCCGCCTCTAGCTCACTCGCTC

Human GTGCGGGTTGGCGGCAGGCGGGGCGCCCCGGCGCGC--CCCGCCTCCGGCTCACTCGCGC

Chimp GTGCGGGCTGGCGGCAGGCGGGGCGCCCCGCCGCGC--CCCGCCTCCGGCTCACTCGCGC

Dog GTGCGGGCTGTCAGCAGGCGGGGGCCCCCAGCGCGC--CCCGCCTCCGGCTCACTCGCGC

Cow GTGCGGGCTGTCAGCAGGCGGGGGCCCCCACCGCGC--CCCGCCTCCGGCTCACTCGCGC

Elephant GTGCGG-CTGTCAGCAGGCGGGGACCCCCACCCAGCCCCCTCCCTC-GGCTCACTCGCCC

Tenrec GTGCGG-CCGGCTGCAGGCGGGGGCCCCCGCCC-GCCCCCTCCCTCCGGCTCCCTGGGGC

CIZF - CAS interating zinc finger protein

ETSF - Ets/Elk family

FKHD - Forkhead

IRFF - Interferon regulatory factors

LEFF - LEF1/TCF

MYOD - Myoblast determining factors

NFAT - Nuclear factor of activated T-cells

NKXH - NKX homeodomain factors

RBPF - Mammalian transcriptional repressor RBP-Jkappa/CBF1

SP1F - GC-Box factors SP1/GC

ZBPF - Zinc binding protein factors

TSS - predicted Transcription start sites ≥ 0.9

**Annotated genomic alignment -500 to +100 of Mouse exon -2b**

>------BRNF-----------> >---GZF1---->

Mouse tttcgtacccattcgaaaaggttttggtatgtttgtttgtacatgtgtgtgagtttatac

Rat actggtacccattttaaaaggttctagtgtgtttgtttgtacatgtgtgtgagtttatac

>---------RX

Mouse acgagtgtgagagatgacggtgatgatgatggtgatggtgatacaaacgtgaagttcaga

Rat atgtgtgagagagatggtggtggtgatgatgatgatgatgatataaacttgaagttcaga

>---MYT1---->

RF----------> >----DICE----->

Mouse ggacagctttggggagtcggttctctccaccatgagagtttggggggttggactcagatc

Rat gaacagctttgaggagtcagttctctccaccatgagagtttgggggattgaactcagatc

<----RREB-----<

Mouse tccagggttggtaacaaacacgttcatccactccagttcctagccagctttttgtttgtt

Rat ttcagggttggtaacaaataatttcatccactccacttcccagtcgg--ttttgtttgtt

>-

Mouse tgtggctttttgggtttttttgttttttgtttttggtttttttgaaacaaggtgttctgg

Rat tgt--------------------------------------ttgagacaaggtgtccggg

<

>-----PARF------>

-----CDXF-------> >-----EVI1------>

Mouse ttagttttatgtcaacttgacacaagctagtgccatcagagaagagggagcctcaataga

Rat ctagttttatgtcaactcgacacaagctagtgtcaccagagaggagggagcctcagttga

------CREB-------<

tss tss

>------SRFF-------> <-> <>

Mouse gaaaatgcctttataaaatctgattctaggcaagcctgcggagcattgtctcaattagtg

Rat gaaaatgcctttataagagctgactctaggcaagcctgtggagcactttcttaattagtg

>-----EKLF------>

Mouse attggtgtgggagtgccccgccccttgtgggtgtggcctatcttgagcggtaagaaagca

Rat atcgatgtgggagggcccagcccactgtgggtgtggcctatcttgagctgtaagaaagca

<-----EVI1------<

>----CAAT----->

tss

<-> |---------------------------- ESTs

tss |------------------------------- ESTs

<> |-------------------- transcript3 / ESTs

|-------------------------------- RefSeq / ESTs

Mouse ggcccagccagccagtcagcAGCATTCCTCCGAGGTCTCTGCATCAACTCCTGCCCTGTG

Rat gactgggcaaaccaatgagcAACATCCTTCTGGGGTCTCTGCATCAGCACCTGCCCTGTT

|---- intron

|---------------------------------------- ESTs

Mouse TGAGTGTCTGTCCTGACTTCCTAAGAAGACGAACAGTGATGTAGAAGTgtaagccaaacc

Rat TGAGTTTCTGCCCCGACTTCCTATGA---TGCACAGTGATGTGGAAGTgtaagccgaatt

Factors conserved in Mouse & Rat, not confirmed in other species

BRNF - Brn POU domain factors

CAAT - CCAAT binding factors

CDXF - Vertebrate caudal related homeodomain protein

CREB - Camp-responsive element binding proteins

DICE - Downstream Immunoglobulin Control Element

EKLF - Basic and erythroid krueppel like factors

EVI1 - EVI1-myleoid transforming protein

GZF1 - GDNF-inducible zinc finger gene 1

MYT1 - MYT1 C2HC zinc finger protein

PARF - PAR/bZIP family

RREB - Ras-responsive element binding protein

RXRF - RXR heterodimer binding sites

SRFF - Serum response element binding factor

TSS - predicted Transcription start sites ≥ 0.9 (none)

tss - predicted Transcription start sites ≥ 0.5 (very poor quality score)

**Annotated genomic alignment -500 to +100 of Mouse exon -2c**

Mouse agtttctgtcctgcctggaagaacgggatgggatgtgggacttctcactcttgcgctacc

Rat agtttctgccctgtctggaaggacgggatgggatatgggaccgctaattcttgccctacc

Human agtttctgcctcgcctggaaggaaaagacggggcatggggcttctcacacttgcactccc

Dog --------------------------------------ggctcctcatgcttgcagtgcc

Cow aatttt--------------------------------ggctcctca--cttgtagcagt

Armadillo tatttctgccttgcccgaaaggggaagac---------ggctcctcttccttgcaccacc

Elephant catttctgccctgcctggaaaggaaggacgcggcacagagcacccctgcaccgca-----

Tenrec catttctgtggagcggagaagggcaggg-agggcatggggctcctctcagccgaggggtc

>-----CHRE--

>-----EGRF---

Mouse tctgtgctcagctcatagtagctgccctgaggtaaccctgcctgtgggcatgtgtgggtg

Rat tctgtgcccagctcaaagtagctgccccgaggtaaccctgcctgtgggcatgtgtgggtg

Human tgggtgctcag-----agtggctgccctgtgataacccttcttatgggcatgcgtgggtg

Dog aggcttctgagcttggtctggctgccctgagttaaccctgcttgtgggcatgtgtgggtg

Cow taggtgttcaacttggtctggctgccctgagttaaccctgcttgtgggcatgtgtgggtg

Armadillo caagcgctctgcttggtctcgctgccccgcgataaccctgcttgtgggcatgtgtgggtg

Elephant ----tgcttcgcttggtccagctgctctgacataaccctgcttgtgggcatgtgtgggtg

Tenrec catgtgccctaagaggtccagctgccctgagataaccctgcttgttggcctgtgtgggtg

<---------RXRF----------< <--------P53F---

<----RREB-----<

<-----EKLF------<

---->

--->

Mouse tcgtggcgaagccgccggccgttgagcagaacaaagatacttgctgtttgctgcacccaa

Rat tcgtggtgaagccactggaggttgagcagaacaaagacacttgctgtttgttacattcga

Human gtgtggtgaagccactccgggttgagcaaaacaaaaa-------tgtttgcagcatttgt

Dog tcgtggtgaagccactcaaggtcgaacaaag-----a-------tgcttactgcatccgc

Cow gcgtggtaag--tgctcaaggttgaacaaagcaaaga-------tgctcactgcatctgt

Armadillo gcgtggcaaagccgctcgaagttgaagtgagcaaaga-------tgctttctgcatccgt

Elephant tactggtaaagctgcattaggtt-----gaacaaaga-------tgctttctgcatccgg

Tenrec tcctggtgaggctgcaccagggtgaacggagcaaaga-------tgctttttgcatccgg

------<

>-MZF1-->

>-------ETSF-------->

Mouse gagatgctacggaagttggctgtctgcagaggggaagttctgctggagaaggctcttggg

Rat gagttgctgcggaagtcggctggctgcagaggggaagttctgcaggagaaaggtcttggg

Human gagaagaagctggagttgcttggctttggaggggaagttctgcaggg--acgttctgcag

Dog gagaagctgcagacgctggatggctagggaggggaagttctgcaggagaagcagcagaag

Cow gagaaaccacgggggctggatggttgcgcaggggaagttctgcaggaagaggggcagaag

Armadillo gagaaggtgcagaggttggctgactttagagggggagttctgcaggagaatgaacagaag

Elephant gccctactgcagaggtt--------tcgagggggacgttctgcaggagaacagacagaag

Tenrec gagat-ctgtgggggtt--------tcgagggggaagttctgccggagagcgagcacaag

Mouse aaccaagggttgcaggcctttgccagcaggctctccacggggcagcccccacccttccgg

Rat aaccaagggt-gcaggtctttgccagcaggctctccacagagcagcgcccagccttccag

Human ga--gagggttacaggcttctgccagcctgttctccactgaccagccccaccctttaatg

Dog gaaacaggtctggaggcgcctgccagcctgttctccaccgaccagc------cctttaat

Cow gaaacaggtctggagacccctgcctccatgttctccactgaccggctcccctcctcaaaa

Armadillo gaacgagaactggaaacacctgccggtctgttctccacggaccagtcccaccctttcgga

Elephant gaagtagaactggaggtccctgacagcctgcct---------------------------

Tenrec gcacgtgcgccaaaggtctctgtcgatctgctctcc------cattccccccctctcacc

|------------------------------------------------ex2d-----

Mouse gctaggacttctttcctctcccatcaaatggggccagagtgcttccggtggtatccacct

Rat gctaggacttcttttctgtcccatcaaa-ggggccagagcgcttccagcagtgcccacct

Human actaacacctcttttctcttccatcaaatggggccaaagtatttccatcagtattaattt

Dog aacagactcttcttcctcttccataaaatggggcggaaggatttccttca----------

Cow aatagaatctcccttcttttccataaaatggggctggaggatttccgtca----------

Armadillo gctgacaccttctttc-cttccataaagtggggccaatgtgtttccaacaggactaattt

Elephant ---actgcgctgggtcccttccagaaaggggggccagagcatttctgtcaggacaagttt

Tenrec cctacagccccttttcccacccataaagtgagtaccaagtatttctgttggg-cacgttt

<-------ETSF--------<

--------------------------------------------|

Mouse ccagagagaaggacccacgtgagctccgagggtggctttgctatggcaagtcctttttgg

Rat ccagagagaagggctggcgt------------------------------ttctctttgg

Human cctgagaaaagagctgatgtaagtaatgcggttgactttcttgcagctaattcttctagg

Dog ---------gggggtcatgtgagtgataatg---tcttccaggcggctaattcttctagg

Cow ---------ggaggtcacgtgagtaacact----tcttccgtaaagcca-ttcttctagg

Armadillo ccggaggattgggctcacgtgagtagtg------actttcttgcagctaattcttccatg

Elephant ccagagcagtgagctcgtgtgagtattacg----cctttcttacagctcacttttccgtt

Tenrec caggagcagtgagctcgggtgaagctcaaa----cctttcttaccaccctttctcccatg

>---GKLF---->

<-tss-> >-----LEFF------>

Mouse gttgcttcagatgacccgccatgagcagttcgttttatttacagttcaaagggaacctga

Rat attgcttcaaattacgtgtcataagcagttcattttatttacagttcaaaggggacctga

Human attgctgtccattctccactgcaactagttcattttttttacaattcaaagggaacttga

Dog actgctataaattctccaccatggctcgtctattttgtttacaattcaaagggaacttga

Cow actgctataaatgctccaccatagctggtccatttcac---cgattcaaaaggaacttga

Armadillo actgctgtaaattctccaccgtaactggctagtttgattgacaatcccaggggaacttga

Elephant agtgtcgtgaattctccaccttaagtggttcattttatttacaattcaaagggaactgga

Tenrec agggttgtgaattccccgccatcgctggcgcatattctgtacaattcaaagagaatggga

<-----FKHD------< <--

<------STAT-------<

<---CHRF----<

>---GATA----> >

>---------NR2F----

>--------MEF2--------->

|-------------------- transcript4 / ESTs

Mouse ggtagcttcctctaccacacACATGACAACAGTTAAAAATAGAGAGAGAGATCAGAAACC

Rat ggtagcttcccctaacacacACATGACAACAGTTAAAAATAGAGTGAGAGATCAGAAACC

Human ggtagcttcctccaaggcacACATGACAACAGTTAAAAATAAAGAGAGAGCTCAGAAACT

Dog agtagcttcctccaaa-cacACATGACAGCAGTTAAAAATAAAGAGAGAGATCAGAAACC

Cow agtagcttcctccaaaacacACATGACAACAGTTAAAAATAAAGAGAGAGATCAGAAACT

Armadillo ggtagccccctccaaaacacACG-GACAGCAGTTAAAAATAAAGAGAGAGATCAGAAACC

Elephant ggcctcttccttcaaaacag--------ACAGTTAAAAATAAAGAGAAAGATCAGAAACC

Tenrec ggttccttagcacacaacaaTCA-GACAACAGTTAAAAATAAAGAGAGAGATCAGAAACT

-----ETSF--------< <---MYBL----<

<------STAT-------< <---HOMF----<

-------ETSF-------->

------> <---tss---> <-tss->

Mouse AGAGAGGGGAAGAAGGCCGGAGGCCATGCTATCGGGAACTTGAGGAATGAGGACGGCTCT

Rat AGAGAGGGGAAGG----CTGAGGCCA--CTATCGGGAACTTGAGGAAAGAGAACAAGTCT

Human GCATAGAGGAAGG----CAGCAGCCGTTCTATCAGGAACTTGAAGAACGATGATGGCTGT

Dog ATAGAGAGGAAGG----CAGAAGGCAGTCTATCGGGAACTTGAGAGACAATGATGACTGT

Cow GTAGAGAGGAAGG----CAGAAG----TCTACCAGGAACTTGAGGGGCTG-------TGT

Armadillo ATATAGAGGAAAG----CAGAAGGCATTCTACCAGGAACTCGAGGCACACTGATGACTGT

Elephant GTATACAGGAAAG----CAAAAGGCATTCTACCAGGAACTTGAGGGACGAGGATGACTTT

Tenrec CTGCAGAGGAAGG----CAGAGGGCATTCTACCAGGAACTTCAGGCACGAGGATGACTGT

CHRE - Carbohydrate response elements

CHRF - Cell cycle regulators: Cell cycle homology element

EGRF - EGR/nerve growth factor induced protein C & related factors

EKLF - Basic and erythroid krueppel like factors

ETSF - Ets family

FKHD - Fork head domain factors

GATA - GATA binding factors

GKLF - Gut-enriched Krueppel like binding factor

HOMF - Homeodomain transcription factors

LEFF - LEF1/TCF

MEF2 - MEF2, myocyte-specific enhancer binding factor

MYBL - Cellular and viral myb-like transcriptional regulators

MZF1 - Myeloid zinc finger 1 factors

NR2F - Nuclear receptor subfamily 2 factors

P53F - p53 tumor suppressor

RREB - Ras-responsive element binding protein

RXRF - RXR heterodimer binding sites

STAT - Signal transducer and activator of transcription

TSS - predicted Transcription start sites ≥ 0.9 (none)

tss - predicted Transcription start sites ≥ 0.5

**Annotated genomic alignment -500 to +100 of Mouse exon -2d**

Mouse gtattctaaaagtaaaaccgtatcattagcattgccaaataacagaaaggaagaccaaag

Rat gtattctaaatgtaatactgtatcgttagcattgccagataacagaaaggaagagggaag

Human ------------------------------------------------------------

Dog ------------------------------------------------------------

Cow ---ttctcaatgttggaagccattatcagcgtccttatttttcagatgaggaaatggagg

Armadillo caattctaaacttgagaat-tgtcattagcatcccccttttacagatgagcaaagagaag

Elephant cggttctcagaattgagagctatgatgagcattcccattttacagatgag--aaaggagg

Tenrec --------------------------------------------------ggaccagagg

Mouse cacagagaggttaagtcacttgcctgaagttgcacagcgccacaactgcagttggctcta

Rat ctcagggaggttaagtcgcttgcctgaagttgcacagcgccacagctgcagtctgctcca

Human -------------------------------------ctccacagtggcagttggttcca

Dog ------------------------------------------------------------

Cow cacagagaggttaagccactcatctgaggtcacacagctccacaggggcagttggctgca

Armadillo cacaaagaggttaagtcacttgtccagggccacacagctccctagtggtggctggctaca

Elephant cacagagaggttaagtcacttgcctaagatcacacagctccatagtgacagttggctcca

Tenrec cacagagaggttaagtcacttgcctgaggtcacacagctctgttggggtcgttggctcca

<------PARF-------<

Mouse caggaggttcttgttctggaccaaaatgcagctatgcttgcccatgctttccttgctcac

Rat caggaggttcttgctctccaccaaaatccagttaagctt-cccgtgcttttcctgttcaa

Human aagaaggt----gctcttaaccc--------ctaagcttcactatacttttcctggccaa

Dog ------------------------------------------------------------

Cow gggaaggt----gctcttaattg--------cgactcttcaccacgctttccttcaccac

Armadillo gggaggat----gcacttgacca--------ctaagtctcaccacgcttttcctggccaa

Elephant ggaagggt----gctcttaactg--------ctaagcctctccaaactttttctggctaa

Tenrec gggaaggt----gatcttctctg--------caaagcctccccactcttttcctggctag

Mouse agggtagggacactgggtagtttctgtcctgcctggaagaacgggatgggatgtgggact

Rat agggtaggggccctgggtagtttctgccctgtctggaaggacgggatgggatatgggacc

Human agggtaaagactc-gggtagtttctgcctcgcctggaaggaaaagacggggcatggggct

Dog --------------------------------------------------------ggct

Cow aggacaagggttt-ggataatttt--------------------------------ggct

Armadillo gcagcaaaggttt-cggttatttctgccttgcccgaaaggggaagac---------ggct

Elephant agggcaagggctt-gggtcatttctgccctgcctggaaaggaaggacgcggcacagagca

Tenrec agggcgagggctg-gggtcatttctgtggagcggagaagggcaggg-agggcatggggct

Mouse tctcactcttgcgctacctctgtgctcagctcatagtagctgccctgaggtaaccctgcc

Rat gctaattcttgccctacctctgtgcccagctcaaagtagctgccccgaggtaaccctgcc

Human tctcacacttgcactccctgggtgctcag-----agtggctgccctgtgataacccttct

Dog cctcatgcttgcagtgccaggcttctgagcttggtctggctgccctgagttaaccctgct

Cow cctca--cttgtagcagttaggtgttcaacttggtctggctgccctgagttaaccctgct

Armadillo cctcttccttgcaccacccaagcgctctgcttggtctcgctgccccgcgataaccctgct

Elephant cccctgcaccgca---------tgcttcgcttggtccagctgctctgacataaccctgct

Tenrec cctctcagccgaggggtccatgtgccctaagaggtccagctgccctgagataaccctgct

<---------RXRF----------<

<-----EKLF----

>-----CHRE------>

>-----EGRF------>

<-tss->

Mouse tgtgggcatgtgtgggtgtcgtggcgaagccgccggccgttgagcagaacaaagatactt

Rat tgtgggcatgtgtgggtgtcgtggtgaagccactggaggttgagcagaacaaagacactt

Human tatgggcatgcgtgggtggtgtggtgaagccactccgggttgagcaaaacaaaaa-----

Dog tgtgggcatgtgtgggtgtcgtggtgaagccactcaaggtcgaacaaag-----a-----

Cow tgtgggcatgtgtgggtggcgtggtaag--tgctcaaggttgaacaaagcaaaga-----

Armadillo tgtgggcatgtgtgggtggcgtggcaaagccgctcgaagttgaagtgagcaaaga-----

Elephant tgtgggcatgtgtgggtgtactggtaaagctgcattaggtt-----gaacaaaga-----

Tenrec tgttggcctgtgtgggtgtcctggtgaggctgcaccagggtgaacggagcaaaga-----

--<

<--------P53F---------<

<----RREB-----<

>-MZF1-->

>-------ETSF------

Mouse gctgtttgctgcacccaagagatgctacggaagttggctgtctgcagaggggaagttctg

Rat gctgtttgttacattcgagagttgctgcggaagtcggctggctgcagaggggaagttctg

Human --tgtttgcagcatttgtgagaagaagctggagttgcttggctttggaggggaagttctg

Dog --tgcttactgcatccgcgagaagctgcagacgctggatggctagggaggggaagttctg

Cow --tgctcactgcatctgtgagaaaccacgggggctggatggttgcgcaggggaagttctg

Armadillo --tgctttctgcatccgtgagaaggtgcagaggttggctgactttagagggggagttctg

Elephant --tgctttctgcatccgggccctactgcagaggtt--------tcgagggggacgttctg

Tenrec --tgctttttgcatccgggagat-ctgtgggggtt--------tcgagggggaagttctg

-->

Mouse ctggagaaggctcttgggaaccaagggttgcaggcctttgccagcaggctctccacgggg

Rat caggagaaaggtcttgggaaccaagggt-gcaggtctttgccagcaggctctccacagag

Human caggg--acgttctgcagga--gagggttacaggcttctgccagcctgttctccactgac

Dog caggagaagcagcagaaggaaacaggtctggaggcgcctgccagcctgttctccaccgac

Cow caggaagaggggcagaaggaaacaggtctggagacccctgcctccatgttctccactgac

Armadillo caggagaatgaacagaaggaacgagaactggaaacacctgccggtctgttctccacggac

Elephant caggagaacagacagaaggaagtagaactggaggtccctgacagcctgcct---------

Tenrec ccggagagcgagcacaaggcacgtgcgccaaaggtctctgtcgatctgctctcc------

|-------------------- transcript5 / ETSs

Mouse cagcccccacccttccgggcTAGGACTTCTTTCCTCTCCCATCAAATGGGGCCAGAGTGC

Rat cagcgcccagccttccaggcTAGGACTTCTTTTCTGTCCCATCAAA-GGGGCCAGAGCGC

Human cagccccaccctttaatgacTAACACCTCTTTTCTCTTCCATCAAATGGGGCCAAAGTAT

Dog cagc------cctttaataaCAGACTCTTCTTCCTCTTCCATAAAATGGGGCGGAAGGAT

Cow cggctcccctcctcaaaaaaTAGAATCTCCCTTCTTTTCCATAAAATGGGGCTGGAGGAT

Armadillo cagtcccaccctttcggagcTGACACCTTCTTTC-CTTCCATAAAGTGGGGCCAATGTGT

Elephant ---------------------ACTGCGCTGGGTCCCTTCCAGAAAGGGGGGCCAGAGCAT

Tenrec cattccccccctctcaccccTACAGCCCCTTTTCCCACCCATAAAGTGAGTACCAAGTAT

<-------E

tss

<->

Mouse TTCCGGTGGTATCCACCTCCAGAGAGAAGGACCCACGTGAGCTCCGAGGGTGGCTTTGCT

Rat TTCCAGCAGTGCCCACCTCCAGAGAGAAGGGCTGGCGT----------------------

Human TTCCATCAGTATTAATTTCCTGAGAAAAGAGCTGATGTAAGTAATGCGGTTGACTTTCTT

Dog TTCCTTCA-------------------GGGGGTCATGTGAGTGATAATG---TCTTCCAG

Cow TTCCGTCA-------------------GGAGGTCACGTGAGTAACACT----TCTTCCGT

Armadillo TTCCAACAGGACTAATTTCCGGAGGATTGGGCTCACGTGAGTAGTG------ACTTTCTT

Elephant TTCTGTCAGGACAAGTTTCCAGAGCAGTGAGCTCGTGTGAGTATTACG----CCTTTCTT

Tenrec TTCTGTTGGG-CACGTTTCAGGAGCAGTGAGCTCGGGTGAAGCTCAAA----CCTTTCTT

TSF--------<

CHRE - Carbohydrate response elements

EGRF - EGR/nerve growth factor induced protein C & related factors

EKLF - Basic and erythroid krueppel like factors

ETSF - Ets family

MZF1 - Myeloid zinc finger 1 factors

P53F - p53 tumor suppressor

PARF - PAR/bZIP family

RREB - Ras-responsive element binding protein

RXRF - RXR heterodimer binding sites

TSS - predicted Transcription start sites ≥ 0.9 (none)

tss - predicted Transcription start sites ≥ 0.5

**Annotated genomic alignment -500 to +100 of Human exon -1**

Human ggagtgggcggcccctcccctcacactcttcctttttatctccagcccaatagggcatga

Chimp ggagtgggcggcccctcccctcacactcttcctttttatctccagcccaatagggcatga

Rhesus ggagtgggcggcctctcccctcacactcttcctttttatctccagcccaataggggatgg

Bushbaby tgaatgggcagcccttgccctcatattcttccttttcatctccagccta--aggggataa

TreeShrew aaagtggatggcctctcccctcacacaatttctttttatctccagctgaacaggg-atac

Mouse --------------ctcttctcacagctgaccttctcatctctaacccagca--g-ttga

Rat --------------ctccgctcccagctgagcttctcatctctaacacaacaagg-gtga

Rabbit --------------cttccctccctgc-aacct-------------acagca----aaga

Dog tgagtggacagcctctccccacacacccatccttttcatctccag-caagcaggg-atga

Cat tgactgggccgcgtctccctgcacgctcactctcttcatctccag-gaagcaggg-atga

Horse tgagtgggcggcccctgcccacacacct-tccttttcatctccat-cccgcaggg-atga

Cow tgagtgggcgacccctccccacacaccattccctttcatctccac-aaagcaagg-ataa

Armadillo cgagtgggtggccccttcccagccgccctttgttttcatttccagc--------------

<---MAZF----< <---GATA----< <------C

<-MZF1--<

<------ETSF---------<

>----NKXH--

>---GATA---->

Human attttcctttagacctgggccttgaaaacagatccacatggggaataaagatttcaagtt

Chimp attttcctttagacctgggcctcgaaaacagatccacatggggaataaagatttcaagtt

Rhesus attttcctttagacctgggccttgaaaacagatctacatggggaataaagatttcaagtt

Bushbaby attaccctttagccttgggccttgaaaacagagccacatggggactcaagacctcaagtt

TreeShrew attttcctttagacataggccttggaaacagacccgcatggagaatcaagatctcaagtt

Mouse attttacttc-gacttggctttggggaatggaagtgcacgaggactgaaaatctgaattc

Rat atctccctttagccttggctctggaaa---gagctacatggggaatgaagacctaaagtg

Rabbit attcacctctccacctgggctggaaac---aggcctggttgggaatcaggactcaagttc

Dog gtctccctgtaaatttagtacttga-------------------gtagggacctcaagtg

Cat gtcttcctgtaaatgtaggacttta-------------------tt-gggacctcaagtt

Horse gccttcctttagatttgggtcttga-------------------at-gggacctcaagtt

Cow atcctcctttagatctgggtgttga-------------------atagagacctcaagtt

Armadillo ----tccttcaaaattggaccttgaagacagaaactgttagggaatcctgatcccaagtt

LOX-------< <----HOXH-----<

<-------IRFF----

---> >-------IRFF-------->

Human caccaacctgctttttgcatccatcgaatggaactcgaacccaggcttggctcagatcaa

Chimp caccaacctgctttttgcatccatcgaatggaactcgaacccaggcttggctcagatcaa

Rhesus caccaacctgcattttgcacccatcgaatggaactcgaacccaggcttggctcagatcaa

Bushbaby cacaaacctattttt--cagctattgaacgg-actcgaacccaggcttggctcagatcaa

TreeShrew catgaacttgctttttgcaaccattgaatggaactcgaacccaggctgggctcagatcaa

Mouse cattctccttttattttcagccacgcaatggaattcaaacttaggcatggctccgatcaa

Rat catgaacttttatcttgaagccacgtaatgggattcgaacttatgcagggctcagatcaa

Rabbit ggagaactgcttt-----agccacggaagggaactcgcgcc----caaggctcggctcaa

Dog caggaatctgctttttgcatccattgaatggaacttgaacccaggcttga----------

Cat caggagtctgctttttgcagccattgagtggaacttgaacccaggctcggctcagatcac

Horse cacgaacctactttttgcagccattgaatggagcttaaacccaggcttggctcacaccac

Cow cacagacatgttttttgcagccattgaatggaactcgaacccaggcttgactcagatcac

Armadillo cacctgcctgcttttcccagcccatgaatgggactggaacccaggcttggctcagatcac

----< <---GATA---

<------CLO

<-----HOXF

<-----H

>---GATA---->

>---------AP1R-

Human ttactttgctttcaaaaaaccaaacaaaaccaacaaacagctctaaaaacgatctgagtc

Chimp ttactttgctttcaaaaaaccaaacaaaaccaacaaacagctctaaaaacgatctgagtc

Rhesus ttactttgctttc-aaaaaccaaacaaaaccaacaaacagctctaaaaacgatctgagtc

Bushbaby ttactttgctttc-aaaaaccaaatgaaaccagaaaacagctttaaaaatgatctgagtc

TreeShrew ttactttgctttc-aaaaac---------ccaacaaacagctctaaaaatgatctgagcc

Mouse ctactgtacttaa-aaaa-----------ccaaccagcagcaataaaaga--------cc

Rat ttactctgcttac-gaaa-----------ccaaccagcagctctaaacgacacctgagcc

Rabbit tcagtttgctttc-aaaa-----------tcaacaaacggctccagaaggagactgagcc

Dog --------------------cacacaaaaccaatgaataactctaaaaatgatgtaagtc

Cat ttactttgctttc--aaaaccaaacaaaagcaacaaataactcgaaaaacgatctgagtc

Horse tcgctgtgctttc-aaaaaccaaacaaaaccaacaaataactctaaaagtgatttgagtc

Cow ttactttgctttc-aaataccaaacaaaaccaacaaacaactctagaaatgatctgaatc

Armadillo ttactttgctttc-aaaaaccaaaggaaaccggcagacagctctaaaaacgatctgagtc

--< <---------AP1R-

X-------<

------<

OXF------<

<----CEBP-----<

>----HAML-----> >--

---------> >-------ETSF--------> >----

Human atctgagtgcacattcctgctccaggcactggaaatgctcctgtggttggtttcagtacc

Chimp atctgagtgcacattcctgctccaggcactggaaatgctcctgtggttggtttcagtacc

Rhesus atctgagtgcacattcctgctccaggcactggaaatgctcctgtggttggtttcagtacc

Bushbaby at-----tgtgtattcctgcttca-gcac-ggacgtgctcctggggttggtttcagtacc

TreeShrew atctgtatgcatattcctgcttcaggccctggaattgctcctgtggtttgcttcagtacc

Mouse atctg--tgtatacccttgcgtca-ggtctggggatacccctgtggtctacttctatgct

Rat atctg--tgtgcatcccaccttca-ggtctgggaatgaccctgtgctttacttctgtgct

Rabbit ctctg--cgtgcattgctgctcag-gcgttgcaggggctcctgggattggcttcagtgcc

Dog atct-----tatgatcttgcttcaggtcctggaaatgctccgatggtttgcttcagtgcc

Cat atctgtatatatattcctgcctcaggcactggaggtgctcctgtggtttgcttcagcagc

Horse atctgtatatctgttcctgcttcaggcactggaaatgctcctgtggtttgcct-agtcct

Cow atctgtatataaattcctgcttcggtcactggaaatgttcctgtggtttgcttcagtacc

Armadillo atctg----tgtattcctgcctcaggccctggaa-cgctcctgtgttttgcctcagtacc

---------< <-------IRFF--------<

--CAAT-----> >-----AP4R------>

-LEFF------> >-------CREB--------> >--

Human accaaagggagctgggggtggcattgacgcagatggcaagaaaggccgatcaagtggaat

Chimp accaaagggagctgggggtggcattgacgcagatggcaagaaaggccgatcaagtggaat

Rhesus accaaagggagctgggggtggcattgacgcagatggcaagaaaggccgatcaagtggaat

Bushbaby accaaagggaaccgggggtggcatccacgcagatggcaggaaaggccgatcaagcagaat

TreeShrew accaaagggagccgggggtggcattggcgcagatggcaagaaaggccgatcaagcagaat

Mouse accaacgggaact-gggctggggttgacacaggtatcgagaaaggcctacccagcaaagt

Rat ggcaaagtggact-ggggtggggttgacacaggtagctagataggttgacccagcaaatt

Rabbit cccaagg-------gagctgcaggtggcattggtgccgcggagggcccatccag------

Dog atc-aaaggggct-ggggtggcattgacacaggtggtgggaaaggcccatcaagcagaat

Cat accggaaggagctggggggggcgtcggcacaggtggttctcaaggctcatcgagcaggat

Horse accgaagagagctgggggtggcattgacacagatggtgagaagggcccacccagcagaat

Cow accaaagagagctgggggtggcattgacacagatggtgaaaaaggcccgtcaagcacaat

Armadillo accaaagggagctgggggtggcggtgatgcagatggcgagaaaggcccgtgaagcagaat

<--------ZBPF---------<

<--------ZBPF---------<

<---------AP1R----------<

<-------PTF1--------<

<---NEUR----<

---HOXC------>

Human gtgattggtttgcaaaaaggagagacctagatgaagcagttgattttgagctaacgtcct

Chimp gtgattggtttgcaaaaaggagagacctagatgaagcagttgattttgagctaatgtcct

Rhesus gtgattggtttgcaaaaaggagagacggagatgaggcagttgattttgagctaacgtcct

Bushbaby gtggctggtttacaaaaaagagagagtgaggtgaagcagtcacttttgagctgaagtcct

TreeShrew gtgattggtttgcaaaaagg-gagactgaggtgaagcatttgcttttgagctaaagtcct

Mouse ggggttgtcttacaagaagg-gaatctgagatgaaatgg---------------------

Rat gggcttggcttacaagaagg-ggatctgagatgaaatgg---------------------

Rabbit ------------------------------------------------------------

Dog gtgattggtttgcaaaatg--ggagcctaggtgaaacagttgctttcaagctaaaaccct

Cat gtgattggtttgcaaaacg--gaagcctaggtgggac-----------agctgaagtcct

Horse gtgattggtttgcaaaaag--gggacctaggtggaacagtcgcttttgagctaaagtcct

Cow gtgactggtttgcaaaaag--ggaaccttggtgaaagagttgcttttgagctgaagtcct

Armadillo gtgactcgtttgcaaaaag--ggaaacgaggtgaaacggtagtgtttgagctaaaatcct

Human gtaagagaatgcacaggtttctgaaattctctcttgttaaaatagttttctttctttctt

Chimp gtaagagaatgcacaggtttctgaaattctctcttgttaaaatagttttctttctttctt

Rhesus ataagagaatgcacaggtttctgaaattctctcttgttaaaatagttttctttctttctt

Bushbaby gtaagagaatgtacaggcttctgaggctctcttttt---aaattatattttttctccccc

TreeShrew gtaggagaatgct-----------------------------------------------

Mouse -------------ctggattctgaaattctgtgttt---aaagcgttctttcctctt---

Rat -------------catgggtctgaacgtctgtgttc---aaagtgttctctcctcct---

Rabbit --------------aggtttcagagtcctcattgtc---aaccggctctctttcctt---

Dog gtgagggaatgctaaggtttgtgagactttctcttttaaaaatcattcattctctttctt

Cat gtaagggaatgc-gaggcttgtgagacttcctctttcaaaaatcattctttctctctctc

Horse gtgagagaatgcaaagccatctgagacattctcttttaacattcttttctttttttcttt

Cow ctaagggaatgtgaaggtttctgagactttctctttttaaagaccttctttctctctctt

Armadillo gtaagagaatgtaaaagtttctgaggttctctc--------------------------t

<-----EVI1------< <---

>---GATA---->

>------SRFF-------> >------CLOX------

|----------------------- transcript1 / RefSeq

|---------------------------------------- ESTs

Human tctttcttttccccaacagATAAGGAGTCAGGCCAGGGCGGGATGACACTCATTGATTCT

Chimp tctttcttttccccaacagATAAGGAGTCAGGCCAGGACGGGATGACACTCATTGATTCT

Rhesus t-tcttttttttccaacagATGAGGAGTCGGGCCAGGGCGGGATGACACTCATTGATTCT

Bushbaby --acccccaccccagacagATAAGGAGTCAGTCCACGGCGGGATGACACTCACTGATTCT

TreeShrew ------------------------------------------------------------

Mouse --tgctttctttcagacatCTAAGGAGCCAGGCAGAAGTGGGATGAAGCCCGCTGATGCC

Rat --ttcttcctttcagacacATAAGGAGCCAGGCAGAAGTGGGATGAAGCTCCCTGATGGC

Rabbit --ttctttccgacagac----AAGGAGTCGGATGAGGGCGGGATGAGCCTCGCTGGCCGT

Dog tcttttttttcccagacagATAAGGAGTCAGGCAGGGGCGGAATGGTGCTCATTGATTCC

Cat tcctttttctcccagacagATAAGGAGTCAGGTGGGGGCGGAATGGTGCTCAATGATTCC

Horse tctttttttt--cagatagATAAGGAGTCAGGCAAGGGCGGAATGACGCTCATTGATTCC

Cow tctttcttcctccagacagATAAGGAGTCAGGCAAGAGTGCATTGATGCTCATTGATTCC

Armadillo ctctcttttcttcagacagATAAGTGGTGAGCCCG--GCGGAATG---------------

--EVI1------< <------CLOX---

<-

tss

-> >-----HOXF------> <->

Human AAAGCATCTTTAATCTGCCAGGCGGAGGGGGCTTTGCTGGTCTTTCTTGGACTATTCCAG

Chimp AAAGCATCTTTAATCTGCCAGGCGGAGGGGGCTTTGCTGGTCTTTCTTGGACTATTCCAG

Rhesus AAAGCATCTTTAATCTGCCAGGCGGAGGGGGCTTTGTTGGTCTTTCTTGGACTATTCCAG

Bushbaby AAAGCATCTTTAATCTGCCAGGAAGAGGAGCTTTTGCTGGTCTTTCTTGGACCATTCCAG

TreeShrew ------------------------------------------------------------

Mouse AAAGTACCTTTAATCTCCCAGGCAGAGGGGCCTTGGCTGGTCCTTCTTGGCCCATACCAA

Rat AAAGCACCTGTAATCTCCCAGGCACAGGGGCCTTGGCTGGTCCTTCTCGGCCCATCCCAA

Rabbit GGAGCTCCCCTCACCCGCCAGGCAGAGGGGCCTTTGCTGGTCTTCCTTGCGCCATCCCGG

Dog AAAGCATCTCTGATCTGCCAGACAGAGGGGCCTTTGCTTTTCTTTCTTGGACCATTCCAG

Cat AAAGCATCTTTGATCTGCCAGACCGAGGGGCCTTTGCTGGCCATTCTTGGACCATTCCAG

Horse AAAGCATCTTTAATCTGCCAGGCAGACAGGCCTTTGCTGGTCTTTCTTGGACCATTCCAG

Cow AAAGCATCTTTAACCTGTCAGGCAGAGGGACCTTTGCTGGT--TTTTTGGACCATTCCAG

Armadillo AAGGCATCTTTAATCTGCCAGGCAGAGGAGCCTTTGCTGGCTTTTCTTGGACCATTCCAG

----< <-----LEFF------<

----EVI1------<

<----HOXH-----<

AP1R - MAF and AP1 related factors

AP4R - AP4 and related proteins

CAAT - CCAAT box binding factors

CEBP - Ccaat/Enhancer Binding Protein

CLOX - CLOX and CLOX homology (CDP) factors

CREB - Camp-responsive element binding proteins

ETSF - Ets family

EVI1 - EVI1-myleoid transforming protein

GATA - GATA binding factors

HAML - Human acute myelogenous leukemia factors

HOXC - HOX - PBX complexes

HOXF - Factors with moderate activity to homeo domain consensus sequence

HOXH - HOX - MEIS1 heterodimers

IRFF - Interferon regulatory factors

MAZF - Myc associated zinc fingers

MZF1 - Myeloid zinc finger 1 factors

NEUR - NeuroD, Beta2, HLH domain

NKXH - NKX homeodomain factors

PTF1 - Pancreas transcription factor 1, heterotrimeric transcription factor

SRFF - Serum response element binding factor

ZBPF - Zinc binding protein factors

TSS - predicted Transcription start sites ≥ 0.9 (none)

tss - predicted Transcription start sites ≥ 0.5

**Annotated genomic alignment -500 to +100 of Human exon -2a**

Human cgaagaagtatattttggggtggcacacgctggtctcccatagtcacattttgggtgtgt

Chimp cgaagaagtatattttggggtggcacacgctagtctcccacagtcacattttgggtgtgt

TreeShrew ------------------------------------------------------------

Mouse ------------------------------------------------------------

Rat ------------------------------------------------------------

Dog ------------------------------------------------------------

Cat ------------------------------------------------------------

Horse ------------------------------------------------------------

Cow ------------------------------------------------------------

Elephant ------------------------------------------------------------

Tenrec ------------------------------------------------------------

Opossum ---------------------------------------------acatttatgaaaggt

Human gtcctgagccccaacactggtcacctcgcaatggcgcttggggtccctgggtgcctagag

Chimp gtcctgagccccaacactggtcacctcacaatggcgcttggggtccctgggtgcctaaag

TreeShrew -----------------------ccttgcagcggtgcaccgggtctttgagtgcctagag

Mouse ------------------------------------------------gagctcctaaga

Rat ------------------------------------------------gagcgctcagga

Dog -------------------------------tggctctgggagtgcccgggtgcctagaa

Cat ------------------------------------------gtccctgggcgccaagag

Horse --------ccctaacgcccagttcctcgcgctggcgctggcgatccccgggtgtctaggg

Cow ------------------------------------------------------------

Elephant --------------------------------------------cctccggtgccttgag

Tenrec ------------------------------------------------------------

Opossum gtcatcacccccttcagaagtcacctctaag--------------ccttagtttccacat

Human ctgcaggttaccccctcttcttctcctgctctcagcctttctcccgctcctgctgaaatt

Chimp ctgcaagatacccccacttct---cctgctttcagcctttctcccgctcctgttgaaatt

TreeShrew ctccaggtttctgtccgtccctctcctcctttcagcctttctcctgccactgtgaaaatt

Mouse cagtagccactcaccctgt-----agtactcttagcttgtcttttggttttgttgttgtt

Rat ctgcagccacccctcctgt-----cgtactcccagacggtcttttgtttttgttgttgtt

Dog ctgcagccaccgcctccctct---ccgccctccaacctttctctggccactgtggaaatt

Cat ctgcagcctccgcctccgtct---ccgccctccagccttcctctggccaccgtggaaatt

Horse ctgcagttcccg------------ccgcccgccagcttttctctggccaccgt-caaatt

Cow ------------------------------------------------------------

Elephant ctccagtcactgtcatc---------cgccctcagacatttcttggcctctgggaaaatt

Tenrec ------------------------tgcgccccc--accttgcttggccactgaagaactt

Opossum ctgtaggttgtgctcccctct---agcctctgcagtttccccccagcatctataacggag

>-------MOKF-------->

Human ctttcctgcgtgaaggcggagcgcttaaaggcgcctaggacctttatttcgttgttttcc

Chimp ctttcctgcgtgaaggcggagcgcttaaaggcgcctaggacctttatttcgttgttttcc

TreeShrew ctttggtgtgtggatgtgaaggttttatgagctctcaggtcctttg----gttgttttcc

Mouse gttttctgagtgactgtggcgatgctcagggctctccagacctttatttggcagtgtgcc

Rat gttttctgagtgactgtggcggtgcctagggctctccggacctttatttggccatggg-c

Dog c-ttggtgtgtgaacgtgggggttttatgggct-ttaggacctttatttggttgtttttc

Cat ctttggtgtgtgaatgt----------tgggct-ttaggacctttatttagttgttttcc

Horse ctttggtgtgtgaatgtgggggttttatgggctcttaggacctttatttggttgttttct

Cow ----------------------------------------------------------ct

Elephant ctttggtgtgtgagtgttggggttttatgggctcctaggacctgtacttggttgttttcc

Tenrec ctcgggtgtatgagtgtggggggtcc-tgggctctcggggtgtgtagttggttgttttcc

Opossum cccagg-----gggtgtgaaagcctggcgggcg--taggaatttcctggggtt-----ct

>---

Human tttgcataaaaagaaaacttgttttgtagaagggcaagcaaggtgcagcttccagaggca

Chimp tttgcataaaaagaaaacttgttttgtagaagggcaagcaaggtgcagcttccagaggca

TreeShrew gttgtataaaaaga---cttgttttctagatgggcaagtga-----accttccagaggca

Mouse tttgcatcaagggaagacttgtttttgaacagagc----------tggggttcgaaggca

Rat tttgcatcaagagaagacttgtttttgaacagtgcgagctt-ctgtggggttcaa-----

Dog tttgtataaaaagaaaacttgttttgaagaagggcaag----atgcagcttccag--aca

Cat tttgtataaaaagaacgcttgttttgaagacgggcgag----atgcagctttcagacaca

Horse tttgtataaaaagaaaatttatttagaagaagagcaag----gtgcagcccccagaggca

Cow tctggatacagataaaacttgttttgaagaagggcaag----gtgtagcttccagaggca

Elephant ttggtataaaaagaaaatttgttttgaagacgggcgaggt--gcagagcttcccg--aaa

Tenrec ttcgtataaaaagaaaacttgttttgaaggcgg-----------------tgccg--aca

Opossum tctggttctgaaga--gcccgcc-------------------------------------

<-----MY

----PTF1-------->

Human cctgggatgatgcccctctagcggtaggcgaactggggagtgtgggcggcgggggaaggc

Chimp cctgggatgatgcccctctagcggtaggcgaactggggggtgtgggcggcgggggaaggc

TreeShrew cctggg-tgatgcccctccagcctcgggcgagctggggggagcgggtggcgagggcaggc

Mouse cctgcggtgctaccctcgcagggcccgctgggcttgg----------ggcggg-gctgga

Rat -ctgcggcgctacctttgcagggcccgctgggcttgg----------ggcggg-gctgga

Dog cctgggatgctgctcctccagccgcagacgggg--ggtggagaggacggggggggctggc

Cat cctgggatgatgcccctccagccgcagacgggg-agggggggagggcggggggtgcagac

Horse cctgggatgatgcccctccagccccagacgggtttggggcgccgcgcgggctgctcagac

Cow cctgggctgacgcccctccagcccgagacggggcaggcgaggcaggcggagggttagaag

Elephant gctgcta-gatacccttccatccacagagggcctggg----------ggcgggaccaggc

Tenrec cctgcga-gatgcccctccagcctcagaggg-gtggg----------ggcggg-cgaagc

Opossum -ctggggtaattccccttgagccccagccaggctggg--gcagcggcggggggagtggat

OD------<

>----NKXH

>----RBPF--

>-----ETSF---

>----NKXH----->

>-----LEFF------>

>------NFAT------->

>-------PTF1--------> >-----FKHD------>

>------GREF-------> >-------IRFF-------->

Human gaggccctggaggacctgttgtttttcctgttgcaagtgaaaaggaaacaaagtgggaag

Chimp gaggccctgggggacctgttgtttttcctgttgcaagtgaaaaggaaacaaagtgggaag

TreeShrew gaggcgcagaaggacctgttgtttttcctgttgcgagtaaaaaggaaacaaagtgggaag

Mouse gaggcg-tagaggatcagttgtttttcctgttatctgtagaaaggaaacaaagtgggaag

Rat gaggcg-taggggaccagttgtttttcctgttatctgtagaaaggaaacaaagtgggaag

Dog gggcggcaggaggacctgttgtttttcctgttgcaagtaaaaaggaaacaaagtgggaag

Cat aggcggcatgaggacctgttgtttttcctgttgcaagtaaaaaggaaacaaagtgggaag

Horse agaccgtcagagaacctgttgtttttcctgttgcaagaaaaaggaaacaaaagtgggaag

Cow aagcggcaggaggacctgctgtttttcctgttgcaagtaaaagggaaacaaagtgggaag

Elephant gac----aagagggccggttcttttccctgttgctagta--aaagaaacaaagtgggaag

Tenrec ggg----tagagg---agctgtttttcctgttaccactg--aaggaaacaga--gggaag

Opossum cagggagagggggactggttgtttttcctgttgcgagcaggaaggcaacaaaataggaag

<--CABL---<

<-------ETSF--------<

<--CIZF---<

----->

--->

-------> <TSS> <TSS->

Human tggagtgtgcgggttggcggcaggcggggcgccccggcgcgccccgcctccctccctcga

Chimp tggagtgtgcgggctggcggcaggcggggcgccccgccgcgccccgcctccctccctcga

TreeShrew tggagtgtgcgggctgtcagcaggc-gggccccccaccgc--cccgcctccctcaaccga

Mouse tggagtgtgcggactgtcagtaggcgg---------------------gctgtcccgctg

Rat tggagtgtgcggactgtcagtaggcgg--------------------ccctgtcccactg

Dog tggagtgtgcgggctgtcagcaggcggggcgccccagcgcgccccgcctccctccctcga

Cat tggagtgtgcgggctgtcagcaggcggggctcccc-------------------------

Horse ttgagtgtgcgggctgtcagccagcggggcgccccaccgcgccccgcctccctccctcga

Cow tggagtgtgcgggctgtcagcaggcggggcgccccaccgcgccccgcctccgtccctcga

Elephant tggggtgtgcgg-ctgtcagcaggcggggcacccc---ccgccccgcccccctccctcga

Tenrec tggggtgtgcgg-ccggctgcaggcggggcgcccc---ccgccccgcctccctccctcta

Opossum ttgagggtgccgggggtgagcaggccggttgcatt-----gcctcgacaccttccctgct

<-----EGRF------<

<TSS->

|------------------------------- transcript2

|------------------------------------- RefSeq

||-----------|---------------------------------------- ESTs

Human ggctcactcgcgcccagcgCAGTCGCTCCGAGCGGCCGCGAGCAGAGCCGCCCAGCCCTG

Chimp ggctcactcgcgcccagcgCAGTCGCTCCGAGCGGCCGCGAGCAGAGCCGCCCAGCCCTG

TreeShrew ggctcactcgcgcccagcgCCGTCGCTCAGAGCGGCCGCGGGCCGAGCCGCCCAGCCCTG

Mouse cgcgc-cccgcctctggctCACTCGCGCCTAGCGGCTG-------------CCAGCTCCG

Rat ctcgc-cccgcctctagctCACTCGCTCCTTGCGGCTG-------------CCAGCTCCG

Dog ggctcactcgcgcccagcgCCCTAGCTCCTAGCGGCCGCGGGCCGAGCCGCCCAGCCCTG

Cat ------------------------------------------------------------

Horse gcctcactcgcgcccagcgCCCTCACTCCAAGCGG-CGCGGGCCGAGCCGCCCAGCCCTG

Cow ggctcactcgcgcccagcgCCGGAGCTCCCAGCGG----------------------CTG

Elephant ggctcactcgcccgcggcaCCCTTGCTCCGAGCGGCCGCGGGCCGAGCCGC--AACCCCA

Tenrec ggctccctggggctcggcaCCCTGGCTCC-AGCGGCCGGCGGCCCAGCCAC--AGCCCCA

Opossum ggctgcctgccgccggtagCCGCCGCTGCTGGCCGCCGCCGGCCGGGCTGTCCCGCCCTG

<---TSS---->

Human CCAGCTGCGCCGGGACGgtaagcgatcgccgctggctgcgtcaggggaggtgggaagagg

Chimp CCAGCTGCGCCGGGACGgtaagcgatcgccgctggctgcgtcaggggaggtgggaagagg

TreeShrew CCA-CTGCGCCGGGACGgtaagcgctcgcctctgatggcgtatggggaggtaggctgggg

Mouse CCGACCCAGCCTGGACGgtaagtgctctccacaggc----gcagaagtgtggagaagagg

Rat CGGGCGCAGCCTGGACGgtaagtgctctctatgggtcatggcagaggtgggaagaagaga

Dog CCAGC-GCGCCGGGACGgtaagcgctcgcctctggctgcgtctggggggtggggaagaga

Cat ------------------------------------------------------------

Horse CCAGC-GCGCCGGGACGgtaagcgctcg-ctctggctgcgtctggggggtggggaagagg

Cow CCAGCTGCGCCCGGAGGgtaagcgctcaccttcggctgagtctggggggtgggggagagg

Elephant CCAGCCGCGCCGGGACGgtaagcgctcgcctttggctgcgtctggggaggtgggaggagg

Tenrec CC-GCCGCGCTGGGATGgtaagcgctcgccttttgttgcttctgggggggtaggaagagg

Opossum CCGGCCTCTCCGGTGCTgtaagctgccgcctcttgtttctcttcgggtgatgggaggagg

CABL - C-abl DNA binding sites

CIZF - CAS interating zinc finger protein

EGRF - EGR/nerve growth factor induced protein C & related factors

ETSF - Ets family

FKHD - Fork head domain factors

GREF - Glucocorticoid responsive and related elements

IRFF - Interferon regulatory factors

LEFF - LEF1/TCF

MOKF - Mouse Krueppel like factor

MYOD - Myoblast determining factorsNuclear factor of activated T-cells

NFAT - Nuclear factor of activated T-cells

NKXH - NKX homeodomain factors

PTF1 - Pancreas transcription factor 1, heterotrimeric transcription factor

RBPF - Mammalian transcriptional repressor RBP-Jkappa/CBF1

TSS - predicted Transcription start sites ≥ 0.9

**Annotated genomic alignment -500 to +100 of Human exon -2b**

>-----AP4R------> >--------ZBPF------

Human gaatgaggatgtgctgagaagccagaaacagcactccctgcccccccacccccgccccca

Chimp gaatgaggatgtgctgagaagccagaaacagcgctccctgccccca-acccccgcctcca

Rhesus gaatgaggatgtgctgagaagccagaaacagcagtccctgcccccc-atccccaccc---

Mouse ----------gtgcagagaatccagaagccgcac-ccctgccct---gcccccactgca-

Rat -----------tgctgataatccggaaagagccccccctgccct---gcccccaccccac

Hedgehog g--------------gagaagcctgtgacagcaccccaccac--------------aa--

Dog gat-------gtgctgagaagccagaaagagtgctcccctcct----gcccccccctccc

Cat aat-------gtgctcagaagccagaaacagtgctccccctcc----gccctcccccccc

Horse gat-------gtgctgagaagccagatacagcacgcccctcct----gccctcccccccc

Cow gat-------gtgctcagaagccagaaacagccctcccccacc----accctcccctccc

Armadillo gat-------gagctgagaagccagaatcctccctccctgcc-----ttccccacccctg

Elephant gat-------gggccgagaaaccagaaacagcctttcctgcc-----ctccccaccccct

<-----EKLF------<

<-----EKLF------<

<-----EGRF------<

<---MAZF----<

<-----EKLF--

>-----LEFF------>

---> >-------IRFF-------->

Human cccaccacgctcagaggcctagaacaaaaacataccacaaatagaaatcaaagcctgaga

Chimp cc-accacgctcagaggcctagaacaaaacc-taccacaaatagaaatcaaagcctgaga

Rhesus ---accgcgctcagaggcctagaacaaaaacatcccacaaatagaaatcaaagcctgaga

Mouse -t-actgtacacacatacctcgaacaaaaacacaccacaaatagacaccaaagtctgaga

Rat cc-atcctacacacagtcctagaacagaaacatgccacaaacagacaccagagtctgaga

Hedgehog --------gctcacaggcctagaacaaaaacacgccacaaatagaaacccaagtctgaga

Dog cc-tgtccgctcacaaggccagaacaaaaacacaccacaaatagaagtcaaagtctgaga

Cat cc-tgtccgctcacaggcccagaacaaaaacacaccacaaatagaaatcaaagtctgaga

Horse ac-tgtgcgctcacaggcccagaacaaaaacacaccacaaatagaaatcaaagactgaga

Cow ac-tgagagctcacaggcccagaacgaaaacacaccacaaatagaaatcaaagcctgaga

Armadillo ac-cctgagctcacaggcctagaacaaaaacagcccacaaatagaaatcagagcctgagg

Elephant ac-tgtgagctcacaggcctaaaacaaaaacagaccacaaatagaaatcaaagtctgaga

----< <-------GREF--------<

>---IKRS---->

>----RBPF----->

Human aagaatcgaggagccacgaccctgccgtgggaaaggctgtgtgaccttggccaggtcact

Chimp aagaatcgaggagccacgaccctgccgtgggaaaggctgtgtgaccttggccagatcact

Rhesus aagaatcgaggagccgtgaccccgccgtgggaaaggctgtgtgaccttggccaagtcact

Mouse aaagagtagggagccctgaccccgctgtggaaaaagctg-gccaccctggccag------

Rat aaagggaaaggaaccctgaccctgctgtgggaaaagctgagtgaccctggtccg------

Hedgehog aagaatcagggagccgtgacctggccgtgggaaagggttcgtggccttggcccgatc-cc

Dog aagaatcagggagccatgacccggccgtgggaaaagctgtatggccttggccaagtcacc

Cat aagaatcaaggagccatgacccagccgtgggaaaggctgtgtggccttggccgagtcgct

Horse aagaaccaaggagccatgacccagccgtgggaaaggctgcgtggccttggccaagtcact

Cow aagaatcaaggagccatgacccagccgtgggaaaggctgtgtggcctcggtcaagtcact

Armadillo aaggatcaaagagccatgacccagctgtgggaaaggctgtgtgaccttggccaagtcacc

Elephant aagaatcaaggagccatgacccagccgtgggaaaggccgtgtgaccttggccaagtc---

<-----EREF------<

<---SF1F----<

<TS>

Human tcacttctctgagcgcacttgcctgtctgtaaaatggggtcatggtctcaaccttgggga

Chimp tcacttctctgagtgcacttgcctgtctgtaaaatggggtcatggtctcaaccttgggtt

Rhesus tcacttctctgagtgcacctgcctgtctgtaaaatggggtaatggtctcaaccttgggga

Mouse tca------tgagcgtctctg---gtctgt---------------tctgcatactgggcg

Rat tca------tgagtgtctctg---gtctgt---------------tctgcacactggaca

Hedgehog tgacttctctgaggctatcctctcctctgtgacctggggtt-------------------

Dog taacttctctgggcctatttgctcatctgtaacctagggtggttggactaaccttgacga

Cat taacttctctgaggctgttggcccgtctgtaaacgggggtgggcgtgctaaccttgagga

Horse taactcctctgagtctatttgctcatctgtaaaatggggtaattgtactaaccctgagga

Cow taacctctctgggtttatttcctcatctgtaagatggggtaaaggtaccaacgttgagga

Armadillo taacctctct-agtctgtttgcgcatctgtaaaatggggtctttttacccacctcgcgga

Elephant --acttctctgagtgggtttgctcatctgtaaaatggggtcattatacttaccttaggga

>-----EVI1------>

Human gctgctgggagaaggtgagctaagatgtcagaggcagggtgcaggtgtgaagttcggtct

Chimp gctgctgggagaaggtgagctaagatgtcagaggcagggtgcaggtgtgaagttcggtct

Rhesus gctgctgggagaaggtgagctaagatgtcagaggccgggtgcaggtgtgaagttcagtct

Mouse gctgc---gtgtggctgaaagaagacgtagtaagacaggtgcttgcgtgatatgtggtcc

Rat gagac---gtgtagctgagatcagatggaataagacagtcgaatttgtgatatgtggtct

Hedgehog ----------gcaggtgcgagcgg---------------------------------tct

Dog gctactatgaacaggtgagagaagatgcaaaatgcagaacacagatgtaatgttg--tct

Cat gccgctgtgaacaggtgagaggagatgtcaagtgcaggacacagatgtagcgtttgctct

Horse g---ttgtgagaaggtgagagaagatgtaaaatgcaggatgcaggtgtgatgtttgatct

Cow gttgtcctgagtaggtgagaaaagatgtgaagtgcaggatgcaggtgtgaagtttgacct

Armadillo ggcgttgggagaaggtgtgacaagaggcgaaatgcagggcctgggcgagctgtttggtca

Elephant gttgttgtaagaaggtgcaataagatgtaaaacacaggccacagatgtgaggtttggtct

<-TSS-> <---TSS---->

Human aaaacaaagcccctcgccacagggtactcgccggactcccagctcgccctaaggctcccc

Chimp aaaacaaagcccctcgccacagggtactggccggactcccagctcgccctaaggctcccc

Rhesus aaaacaaagcccctcgcagcagggtcctcgccggactccccgcttgccct-gggctcccc

Mouse caaataaaggccctcaccacagggctttg------------------cctaaccccactg

Rat caaataaaggcccccaccacagggttttg------------------cctaagcccactg

Hedgehog aatagaaagccctccaccac----tccctgctggacgctccca-----------------

Dog aaagcaaagccccttacagcagggtctttattggactctcccattggcaccgcccccctg

Cat caag----------------agggtctttcctggactctcccaccagcacccccacccca

Horse aaaataaagcccctcacagcagggtcctgactggactctcccctggccgcccc----cta

Cow ggaatgaagcccctcacgcaagggtccttaccagattgtctcctcacccccac----ctc

Armadillo aaaataaa--ccctccccgcggggtccaggctggcctctgccctcgccccg------ccc

Elephant aaattaaagcccctcctggcgccatacctgctggactctcccctcgtcccg------ccc

<---

<-TSS->

>---NFKB----> >------STAT------->

Human cacccctgcagtcgggccgggaccgcccctgagcacttccccgtaaggctgcggtgcacg

Chimp gacccctgcagtcgggccgggaccgcccctgagcacttccccgtaaggctgcggtgcacg

Rhesus cgcccccgcagtcagaccgggactgcccctgaccactttcccgtaaggctgcggtggacg

Mouse cacacccacggtcagactccggctgccccagctctctctcctatggggctgtggtaaggg

Rat catacccacagtcagacaagggatgccccagttccccttcctatggggctgtggtaaggg

Hedgehog ----gccacagtcgggaggggactgccc-------ttttcctgtcaagctgc---tgcag

Dog tgcccccacgttcgggctggggctgcccctcaccactttcctgtaaggctgcggtggaag

Cat cgcccccacagccgggctggggctgcccctcgccactttcctgtaaggctgcggtggaa-

Horse cgcccccgcagtggggctggggctgcccctcaccactttcccgtaaggctggggtggaag

Cow cgccaacgacgctgggctggggctgcccctcaccattttcctgtaaggccgcggtggcag

Armadillo tgcccccgcagccgggcaggggctgcccctcgccgctttcctgtaaagcggcggtgggag

Elephant cgcccccgcagctggctgggggcagcccctcaccactttcctgtaatgctgtggtggggg

--EGRF------< <---NFKB----< <------STAT-------<

<-------ETSF--------<

TSS

<--TSS--> <->

Human ggctggggagcacttctgcaccctcgctctccacttcctgtttttctccccaacgaattt

Chimp ggctggggagcacttctgcaccctcgctctccacttcctgtttttctccccaacgaattt

Rhesus ggctggggagcgcttctgcagtcttgctctccacttcctgtttttctccccaacggattt

Mouse agctgggaa---ctcctggacccctgctctcctcttcccgtttctctcctcaacgggttt

Rat agccgggaa---cttctgcacccctgctttccacttcctgtttttctccccaaagaattt

Hedgehog gggcggg-------cctgccggct---cctccacttcctgtttttctccccaacgaatct

Dog ggctgggtagcgcctctgcagccttgctctccacttcctgtttttctccccaacgaattt

Cat ggccggggagcgtttctgcagccttgctctccacttcctgtttttctccccaacgaattt

Horse ggccgggtagcgcttctgcaggcttgctctccacttcctgtttttctccccaacgaattt

Cow ggtggggtagagcttctgcagccgagctctccacttcctgtttttctccccaacgaattt

Armadillo ggctcggtggcgcttctgcaggctcgctctccacttcctgtttttctccccaacgaattt

Elephant ggttgggtagcgcttctgcagccttgctctccacttcctgtttttctcccgaacgaattt

<-------ETSF--------< <-----E

<-----E2FF------<

>-----EGRF------>

>-----EKLF------>

Human tgcgccattactctttcatggggtgaggggggagtgggggcggaggcggaggcctgcctg

Chimp tgcgccattactctttcatggggtgaggggggagtgggggcggaggcggaggcctgcctg

Rhesus tgcgccattactctttcatggggtgaggggcgagtgggggtggaggcggaggcctgcctg

Mouse tgccccgttctttaatcaaggg-----tggaaagtgaaggtgaaggcca--------cta

Rat tgccccgttcttttatcatgggaccgggggggagtgaaagtgaaggccg--------ctg

Hedgehog tataccatagctctttcatca--------gggtgggggggggaggtcaga-----gcatg

Dog tgcaccattgctctttcatcagagcaggggggtggtgaggtaaaggcagaggcctgcctg

Cat tgcaccattgccctttcatcagcgcacgggggtggagaggtgaaggtgaaggccggcccg

Horse tgcaccatggctctttcatcagagagagggggtggtgaggggaaggtggaggccggcct-

Cow tgcaccattgctctttcatcaggccgtgggggtggtg-ggtgaaggcggaggcctgctg-

Armadillo tgcaccattgctctttcat------acagctgggggtcggcgaaggcagggg----cctg

Elephant tgcaccatcgcgctttcat-----------------------------------------

2FF------< <--------ZBPF---------<

|------------------------ transcript3 / ESTs

Human ggaaggaagcccgggggtcaGAGGCGCCCAGGACACCACGAGGTTGGGAGAGGAGTGTGC

Chimp ggaaggaagcccgggggtcaGAGGCGCCCAGGACACCACGAGGTTGGGAGAGGAGTGTGC

Rhesus ggaaggaagccctggggtcgGAGGCGCCCCGGACGCCACGAGGTTGTGAGAGGAGTGTGC

Mouse gggacgaagcctagggctcaGAGAAGCC-AGACCATCACAAAGCTGGCTAGAGAGGCTAC

Rat gggaggaagccaaggagtcaGAGGAGCCTGGGCTATCACAAGACTGGAGAGAGAGGCTAC

Hedgehog agagaacacactgca-------------------------CAGCTAGGAGAGGAGTGTGT

Dog ggaagaaaccctgggggtcaGAGGATCCCAGGGCACCAGGAAG-----------------

Cat ggaaggaaccctggggggcgGAGGATCCCAGGGCCTCAGGAAGTGGGGAGAGCAGTGTAT

Horse ggaaggaaacccgggggtcaGAGGAACCTGGGGCTTCACAAGGTGGGGAGAGGGGTGTGC

Cow ggaaggcgaactgggg------------------------AAGAGGAAAGAGGAGTGTAC

Armadillo gggaggaaacccgg--------GGTACACAGGGCTTCGCGGGGTGGGAAGAGGGGTGTGC

Elephant ------------------------------------------------------------

Human TTTGCACTCTGCACTCTCCAGCTCAGAGTAGCTGAGGATGCTAGGGAGACTGGGGGCTGG

Chimp TTTGCACTCTGCACTCTCCAGCTCAGAGTAGCTGAGGATGCTAGGGAGACTGGGGGCTGG

Rhesus TTTGCACTCTGCAGGCTCCAGCTCAGAGTAGCTGAGGATGCTAAGGAGGCTGGGGGCTGG

Mouse TGGGCCCTCCACGGCCTC------------------------------------------

Rat GAGG--CTACAGGGCTTC--CTCCAGGGCCTCTCACCAAGATAGTAAGTCTAAGAACTGA

Hedgehog CGCA-------AGACCACCAGCTAGAGCCCG---TGGGGGC------GCCTGTGGGCTGC

Dog -CTGGACTC--TGATCTCCGGCTCAGAGCAGCAGTGGAGGCT-GGAGGCTGGTGGTCCAC

Cat TCTGGGCTCTGTGGTCTCCAGCTCGGAGCGGCAGCGGAGGTTGGGAGGCTGGTGGGCTGC

Horse TCTGGACTC--TGGTCTCCAGCTCAGAGTAGCAGTGGAGACCAGGAGGCCGGTGGCCTGC

Cow TCTGGACTCTGTGGGCTCCAACT-------GC--TAGGGGCTGGGAGGCTGGTGGGCTGC

Armadillo GGAA-GCTTTGGGATCCC------------GCCAGTGGTGCTGGGAGGTGGGG---CTGC

Elephant ------------------------------------------------------------

AP4R - AP4 and related proteins

E2FF - E2F-myc activator/cell cycle regulator

EGRF - EGR/nerve growth factor induced protein C & related factors

EKLF - Basic and erythroid krueppel like factors

EREF - Estrogen response elements

ETSF - Ets family

EVI1 - EVI1-myleoid transforming protein

GREF - Glucocorticoid responsive and related elements

IKRS - Ikaros zinc finger family

IRFF - Interferon regulatory factors

LEFF - LEF1/TCF

MAZF - Myeloid zinc finger 1 factors

NFKB - Nuclear factor kappa B/c-rel

RBPF - Mammalian transcriptional repressor RBP-Jkappa/CBF1

SF1F - Vertebrate steroidogenic factor

STAT - Signal transducer and activator of transcription

ZBPF - Zinc binding protein factors

TSS - predicted Transcription start sites ≥ 0.9
